# Integrated single-cell RNA sequencing reveals the tumor heterogeneity and microenvironment landscape during liver metastasis in adenocarcinoma of esophagogastric junction

**DOI:** 10.3389/fimmu.2024.1484234

**Published:** 2025-01-09

**Authors:** Junrui Xu, Ussama Sadiq, Wangruizhi Zhao, Hengbo Xia, Yiwei Liu, Renquan Zhang, Aman Xu

**Affiliations:** ^1^ Department of General Surgery, First Affiliated Hospital of Anhui Medical University, Hefei, China; ^2^ Department of Thoracic Surgery, First Affiliated Hospital of Anhui Medical University, Hefei, China

**Keywords:** single-cell RNA sequencing, adenocarcinoma of esophagogastric junction, liver metastasis, tumor heterogeneity, tumor microenvironment

## Abstract

**Background:**

Adenocarcinoma of the esophagogastric junction (AEGJ) is a highly aggressive tumor that frequently metastasizes to the liver. Understanding the cellular and molecular mechanisms that drive this process is essential for developing effective therapies.

**Methods:**

We employed single-cell RNA sequencing to analyze the tumor heterogeneity and microenvironmental landscape in patients with AEGJ liver metastases. This approach enabled us to characterize the diverse cell populations involved in the liver metastatic process.

**Results:**

Our analysis revealed a significant involvement of fibroblasts and mural cells in AEGJ liver metastasis. We identified a specific fibroblast type in AEGJ liver metastasis and observed distinct gene expression patterns between adenocarcinoma of the esophagogastric junction and other stomach adenocarcinomas. Our study demonstrated high expression of the SFRP2 gene in pericyte cells during the liver metastasis of AEGJ. The incorporation of GEO, TCGA, and immunofluorescence staining of SFRP2 expression enhanced our study. High expression of SFRP2 in pericytes may influence vascular stability and angiogenesis through the Wnt pathway.

**Conclusion:**

Our study provides novel insights into the cellular interactions and molecular mechanisms that underlie AEGJ liver metastasis. Targeting the identified subtype of fibroblasts or influencing SFRP2 gene expression in pericytes may offer new therapeutic strategies for combating this aggressive tumor.

## 1 Introduction

Adenocarcinoma of the esophagogastric junction (AEGJ) is a malignant tumor located at the junction of the lower esophagus and the gastric cardia. Although AEGJ is commonly classified with stomach adenocarcinoma (STAD) in clinical trials (1), it differs significantly from STAD in terms of epidemiology, etiology, and pathological characteristics (2–4). Globally, the incidence of AEGJ is increasing annually, whereas the incidence of STAD in other regions is significantly decreasing (5–8). Due to its specific anatomical location and unique biological characteristics, AEGJ exhibits higher malignancy and a worse prognosis (9).

The liver is the most common site of hematogenous metastasis in gastric adenocarcinoma (10). Liver metastasis is a significant cause of morbidity and mortality in gastric cancer. A population-based cohort study in the US and China indicates that AEGJ is more prone to liver metastasis than STAD in other regions (21.4% vs. 11.8%, p < 0.05) (11, 12). This may be related to its anatomical proximity to the liver’s blood supply and its greater proliferative and invasive capabilities (13, 14). This research also indicates that, compared to distal STAD, AEGJ patients are often diagnosed at a later stage of the disease, experience earlier liver metastasis, and have poorer prognoses (15). Although researchers generally believe that “the unique biological characteristics of AEGJ make it more likely to metastasize to the liver compared to STAD in other regions,” the underlying mechanisms of AEGJ liver metastasis remain insufficiently elucidated, limiting innovations in new treatment methods to improve the prognosis of AEGJ patients.

Recently, single-cell RNA sequencing (scRNA-seq) has become widely used to investigate the cellular and molecular mechanisms of malignant tumors (16–18). By identifying critical cell subtypes and cell-cell interactions, scRNA-seq enables a detailed analysis of the evolutionary trajectories of tumor cells during growth and metastasis, thereby uncovering key molecular drivers and metastasis-related signaling pathways (19, 20). Previous scRNA-seq studies on STAD have reported the developmental trajectories of STAD cells undergoing peritoneal, lymph node, and liver metastases, identifying subtypes with varying malignant biological behaviors (21–23). Studies have shown that specific cell subtypes play crucial roles in shaping the tumor microenvironment of STAD, influencing tumor growth and metastasis (24). However, to date, few studies have reported differences in metastasis-related single-cell transcriptomes among STAD from different regions or explored the potential cellular and molecular mechanisms of AEGJ liver metastasis at the single-cell level.

In this study, we aim to utilize scRNA-seq to thoroughly explore the molecular characteristics of AEGJ liver metastasis, identify cell populations associated with liver metastasis and their functional states, and investigate potential early detection biomarkers and novel therapeutic targets. By enhancing our understanding of the AEGJ liver metastasis process, we hope to contribute to the development of more effective therapeutic strategies, ultimately improving the prognosis and quality of life for patients with AEGJ.

## 2 Materials and methods

### 2.1 Patient and sample collection

This study included 12 cases of AEGJ patients diagnosed at the Departments of General Surgery and Thoracic Surgery at the First Affiliated Hospital of Anhui Medical University. Detailed communications were conducted with all patients or their guardians prior to the experiment, and all provided written informed consent for sample collection and data analysis. All experimental procedures were approved by the Ethics Committee of the First Affiliated Hospital of Anhui Medical University. Based on the NCCN guidelines and several studies conducted in Japan and Europe (8, 25, 26), we established the criteria for resectable AEGJ liver metastasis: the depth of tumor invasion at the AEGJ primary site must be ≤T4a stage, lymph node metastasis must be within the scope of D2 lymph node dissection, and the size of a single metastatic lymph node must be ≤3 cm. The patient must be in an oligometastatic state with one to three metastases confined to the same liver lobe, with no invasion of the hepatic portal or major blood vessels. Additionally, the patient must be able to tolerate hepatectomy, as assessed by liver reserve function. Following rigorous preoperative assessment and the acquisition of written informed consent, two cases of operable AEGJ patients with liver metastasis were included. Postoperative primary AEGJ tissue samples and adjacent normal tissue samples were collected for single-cell RNA sequencing (scRNA-seq). Additionally, postoperative primary AEGJ samples and adjacent normal tissue samples were collected from another ten patients. The collected specimens were stored in liquid nitrogen and fixed in formalin-fixed paraffin-embedded (FFPE) for subsequent experimental analyses.

### 2.2 Tissue dissociation and single-cell suspension preparation

Fresh tumor and normal tissues were cut into approximately 2-3 mm³ pieces in DMEM medium containing 10% fetal bovine serum (SH30406.05; HYCLONE). The tissues were transferred to a gentleMACS C tube (130-096-334; Miltenyi Biotec) containing an enzyme solution (130-095-929; Miltenyi Biotec), tightened, and digested at 37°C in a tissue processor (130-096-427; Miltenyi Biotec). The mixture was then filtered through a 40 μm cell strainer (352340; BD) to collect the cell suspension. After centrifugation of the filtered cell suspension, the supernatant was discarded, and red blood cells were lysed using a red blood cell lysis solution (8570396; QIAGEN). Dead cells and debris were removed using the Dead Cell Removal Kit (130-090-101; Miltenyi Biotec). The cells were washed with PBS (10010-031; GIBCO) and resuspended in an appropriate volume. A hemocytometer was then used to count and assess the cells, resulting in a single-cell suspension.

### 2.3 Library construction and single-cell sequencing

Following the manufacturer’s instructions, single-cell resolution immunome measurements and gene expression analyses were performed using the MGI1SEQ-2000RS high-throughput sequencing kit (1000012554; MGI). In brief, the prepared single-cell suspension was adjusted to a cell density of 1000 cells/μL. Ten microliters of the cell suspension were placed into a 200 μL PCR tube, followed by the addition of 10 μL of 0.4% trypan blue dye. The treated cell sample was then added to a CYTO C-Chip hemocytometer for microscopic examination to calculate cell concentration, viability, and clumping rate. Qualified samples should exhibit an ideal cell viability greater than 90%, with a minimum of no less than 80%, and a clumping rate not exceeding 15%. The Next GEM Chip G was loaded, and an appropriate volume of cell suspension with a concentration of approximately 1000 cells/µL was added to each channel, ensuring that the number of cells per sample was no less than 10,000. The mixture was then further combined with barcode gel beads on the Chromium Controller (10x Genomics). After the reverse transcription reaction, cDNA amplification was performed for 11 cycles using a thermal cycler (S100; Bio-Rad, USA). Using the amplified cDNA as a template, TCR fragments were subsequently enriched. The sequencing libraries for cDNA and TCR were constructed separately according to the manufacturer’s instructions. These libraries were sequenced using a gene sequencer (MGISEQ-2000; MGI) with a paired-end sequencing strategy of 150 bp.

### 2.4 Retrieval and process of scRNAseq data

A unique molecular identifier (UMI) count matrix from public single-cell RNA-seq data of two adjacent non-tumor normal samples (NC) and five tumor samples (two AEGJ, two Body, one Antrum) in 24 STAD patients was downloaded from GSE206785 (https://www.ncbi.nlm.nih.gov/geo/query/acc.cgi?acc=GSE206785). The UMI count matrix was converted into a Seurat object using the R package Seurat (version 4.0.4) (27). Cells with UMI counts < 500, fewer than 200 detected genes, or more than 10% mitochondrial-derived UMI counts were classified as low-quality cells and subsequently removed. Genes detected in fewer than five cells were removed for downstream analyses (https://ngdc.cncb.ac.cn/gsa-human/browse/HRA002336).

### 2.5 scRNA-seq data preprocessing

After quality control, the UMI count matrix was log-normalized. The top 2,000 variable genes were then used to create potential anchors using the Find Integration Anchors function in Seurat. Subsequently, the Integrate Data function was employed to integrate the data. To reduce the dimensionality of the single-nucleus RNA sequencing (snRNA-seq) dataset, principal component analysis (PCA) was performed on the integrated data matrix. Using the Elbow Plot function in Seurat, the top 50 principal components (PCs) were utilized for downstream analysis. The main cell clusters were identified using the Find Clusters function provided by Seurat, with the resolution set to default (res = 0.6). Finally, the cells were clustered into 10 major cell types. Finally, the cells were clustered into 10 major cell types. To identify the cell type for each cluster, gene markers were detected for each cell cluster using the Find Markers function in the Seurat package (v4.3.0). Cell types were then annotated using “ScType” tools (28) with previously published marker genes (29). Additionally, “CellCall”revealed integrated intercellular communication networks, combining ligand-receptor dialogue and intracellular transcription factor dynamics.

### 2.6 Comparison dendrograms

To conduct a phylogenetic analysis of the various cell subpopulations within the scRNA-seq dataset, the BuildClusterTree function from the Seurat R package was utilized. To visualize our results, the ggtree R package was employed (30).

### 2.7 Differential gene expression analysis

Differentially expressed genes (DEGs) were identified using the Find Markers/Find All Markers function from the Seurat package (one-tailed Wilcoxon rank sum test; p-values were adjusted for multiple testing using the Bonferroni correction). For the computation of DEGs, all genes were examined for an expression difference of at least 0.2 on a natural log scale, with an adjusted p-value of less than 0.05.

### 2.8 Pseudotime trajectory analysis by monocle2

Monocle2 (v2.26.0) (31) was used on epithelial cells to uncover the pseudotime trajectory. Dimensionality reduction and trajectory analysis were conducted using the standard workflow with default parameters.

### 2.9 Retrieval and process of TCGA data

The TCGA-STAD project data for 26 distal metastasis samples and 5 lymphatic metastasis samples, including gene expression profiles and clinical information, were downloaded from the GDC database (https://portal.gdc.cancer.gov/projects).

### 2.10 Reads alignment and differentially expressed gene analysis

Gene expression levels were evaluated using FPKM (fragments per kilobase of exon per million fragments mapped). DESeq2 (v1.30.1) software was used to perform differential gene expression analysis using the read count file (32). DESeq2 was also used to analyze differential expression between two or more samples, determining whether a gene was differentially expressed by calculating the fold change (FC) and p-value. Two important parameters were defined: 1) FC: fold change, representing the absolute ratio of expression change; 2) P-value: p-value. The criteria for significant differential expression were as follows: FC ≥ 1.5 or ≤ 1/1.5, and p-value ≤ 0.05.

### 2.11 Survival analysis

Kaplan-Meier survival analysis was conducted using the survival package, with optimal cutoff values for different expression cohorts (high or low) determined using the R package “survminer” (the minimum proportion of high or low expression groups should not be less than 0.3). A log-rank test was performed using the survfit function to evaluate the significance of the high and low expression groups.

### 2.12 Functional enrichment analysis

Protein-protein interaction data often include biologically implausible interactions (i.e., those that are difficult to occur in living cells). The comPPI database (https://comppi.linkgroup.hu/) was used to filter out interacting proteins lacking mutual subcellular localization and to identify proteins that interact with genes of interest. A novel quantitative metric, termed the interaction score, is introduced to reflect the likelihood of interaction.

Gene set variation analysis (GSVA) calculates the variation score for specific gene sets in each sample using the expression matrix, without requiring prior analysis of differences between samples. Transforming genes into pathways enhances the biological significance of the data and makes it more interpretable for life phenomena. The CancerSEA database collates 14 different functional states of tumor cells (33). The Z-score algorithm, proposed by Lee et al., integrates characteristic gene expression to reflect the activity of a given pathway (34). Fourteen functional state gene sets are calculated using the Z-score algorithm in the R package GSVA. The values of each gene set are reported separately as Z-scores. Pearson correlations between genes and the Z-scores of each gene set are calculated.

Download the protein expression data from the reversed-phase protein array of the Cancer Proteome Atlas (TCPA) database (https://www.tcpaportal.org/tcpa/). Pathway activity scores are calculated for ten cancer-related pathways (TSC/mTOR, RTK, RAS/MAPK, PI3K/AKT, hormone ER, hormone AR, EMT, DNA damage response, cell cycle, and apoptosis) based on published findings. The Spearman correlation and P-value between the target gene and pathway activity score are calculated using the cor test function.

To identify functional categories of genes, Gene Ontology (GO) terms and Kyoto Encyclopedia of Genes and Genomes (KEGG) pathways were determined using KOBAS 2.0 (http://bioinfo.org/kobas) (35). The hypergeometric test and Benjamini-Hochberg false discovery rate (FDR) controlling procedure were used to define the enrichment of each term.

### 2.13 Spatial transcriptomics data analysis

To explore differentially expressed genes (DEGs) in the spatially specific tumor microenvironment of STAD, we employed 10×Genomics spatial transcriptome (ST) technology using the GSE203612 cohort (36). The results were analyzed and visualized to determine the expression levels and spatial distribution of JUND, IL24, and SFRP2. Spatial transcriptomics data were processed using the R package Seurat (v4.0.4), with functions such as RunPCA, FindNeighbors, and FindClusters applied to cluster similar spatial transcriptome (ST) points and perform dimensionality reduction. An unsupervised clustering analysis of the scRNA-seq data, in combination with hematoxylin and eosin (H&E) staining sections, was performed to provide initial annotations for distinct clusters. Further annotations were made using cell markers. Based on the annotation outcomes, the cell type with the highest content in each micro-region was identified. The SpatialDimPlot function in Seurat was used to visualize the predominant cell composition in each micro-region. To generate enrichment score matrices, we used the get enrichment matrix and enrichment analysis functions from the Cottrazm software package. The SpatialFeaturePlot function in Seurat was then applied to visualize the expression landscapes of genes in each micro-region and calculate the enrichment score for each cell type. Higher enrichment scores were represented by darker colors, indicating a higher content of that cell type in the spot. Finally, Spearman correlation analysis was performed to assess the correlations between cell contents across all spots, as well as between cell contents and gene expression levels. All analysis and visualization steps were conducted in R software, with public online databases (https://grswsci.top/#shareTabContent) used for additional resources.

### 2.14 Immunofluorescence Staining

To identify fibroblast and pericyte marker genes associated with AEGJ liver metastasis, we conducted immunofluorescence staining for IL24, JUND, and SFRP2 on AEGJ tumor and normal samples. Continuous sections (4 μm thick) of tumor and normal tissues from formalin-fixed, paraffin-embedded samples were stained according to a standard protocol. The tissue sections were deparaffinized and subjected to heat-induced antigen retrieval in citrate buffer at pH 6.0 for 15 minutes. After blocking with goat serum (1:10, Elabscience, E-IR-R111), antibodies were applied: anti-DCN (mouse, 1:1000, Proteintech, Ag6275, lot: 66847-1-Ig) for fibroblast staining and anti-ACTA2 (rabbit, 1:100, HUABIO, ET1607-53) for pericyte staining. The following antibodies were used to detect the respective proteins: anti-IL24 (rabbit, 1:100, HUABIO, ER1911-33), anti-JUND (rabbit, 1:50, HUABIO, ET1612-92), and anti-SFRP2 (mouse, 1:750, Proteintech, Ag18840, lot: 66328-1-Ig). After incubation overnight at 4°C, the sections were washed three times with PBS and labeled with secondary antibodies tagged with FITC (495 nm, 1:50, Elabscience, E-AB-1015) and Cyanine3 (554 nm, 1:50, Elabscience, E-AB-1010). DAPI was used for nuclear staining. Images were acquired using a Leica DMi8S confocal laser scanning microscope.

## 3 Results

### 3.1 Single-cell transcriptome profile of AEGJ with liver metastasis

**Figure 1:**
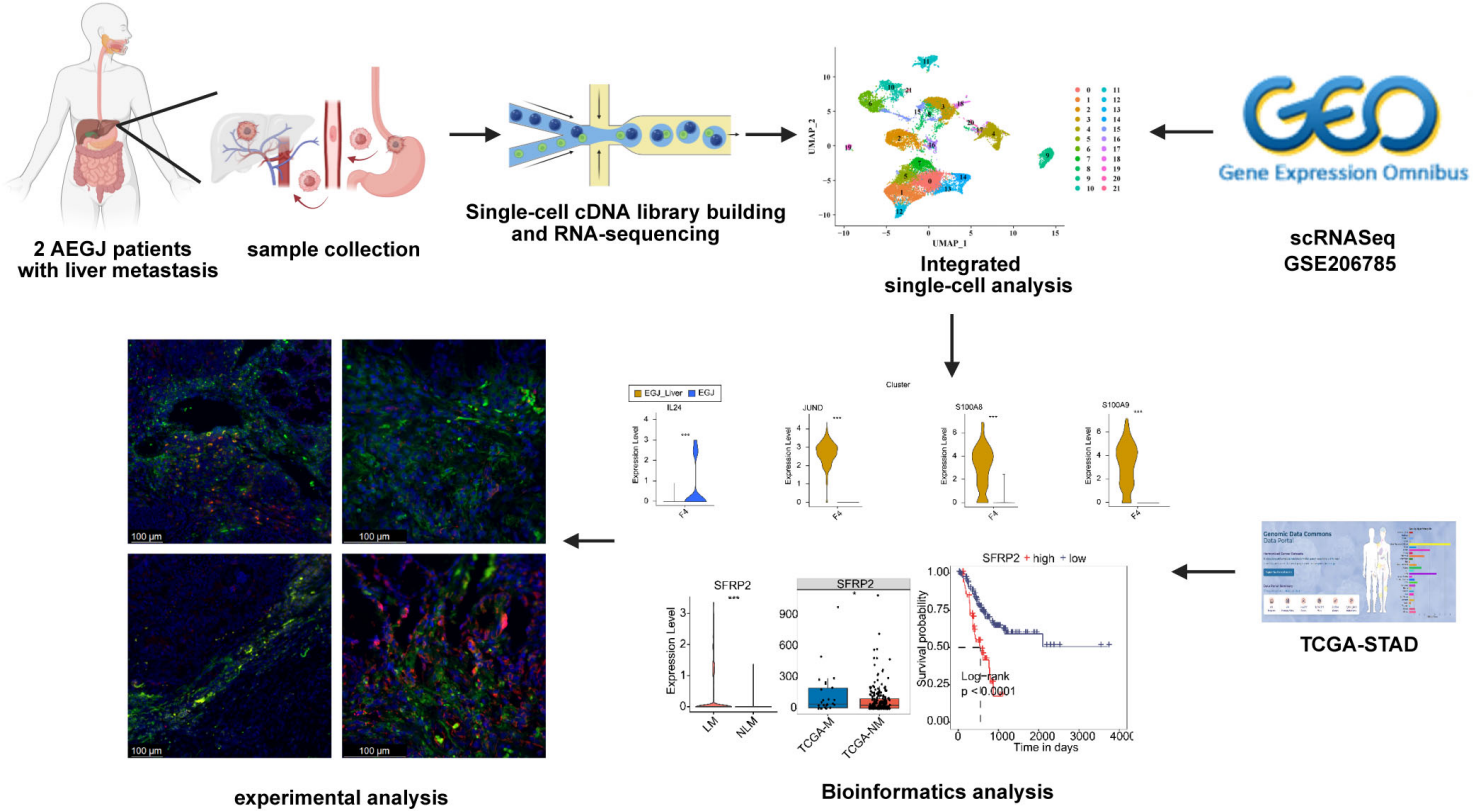
The structured workflow of this study.

**Figure 2:**
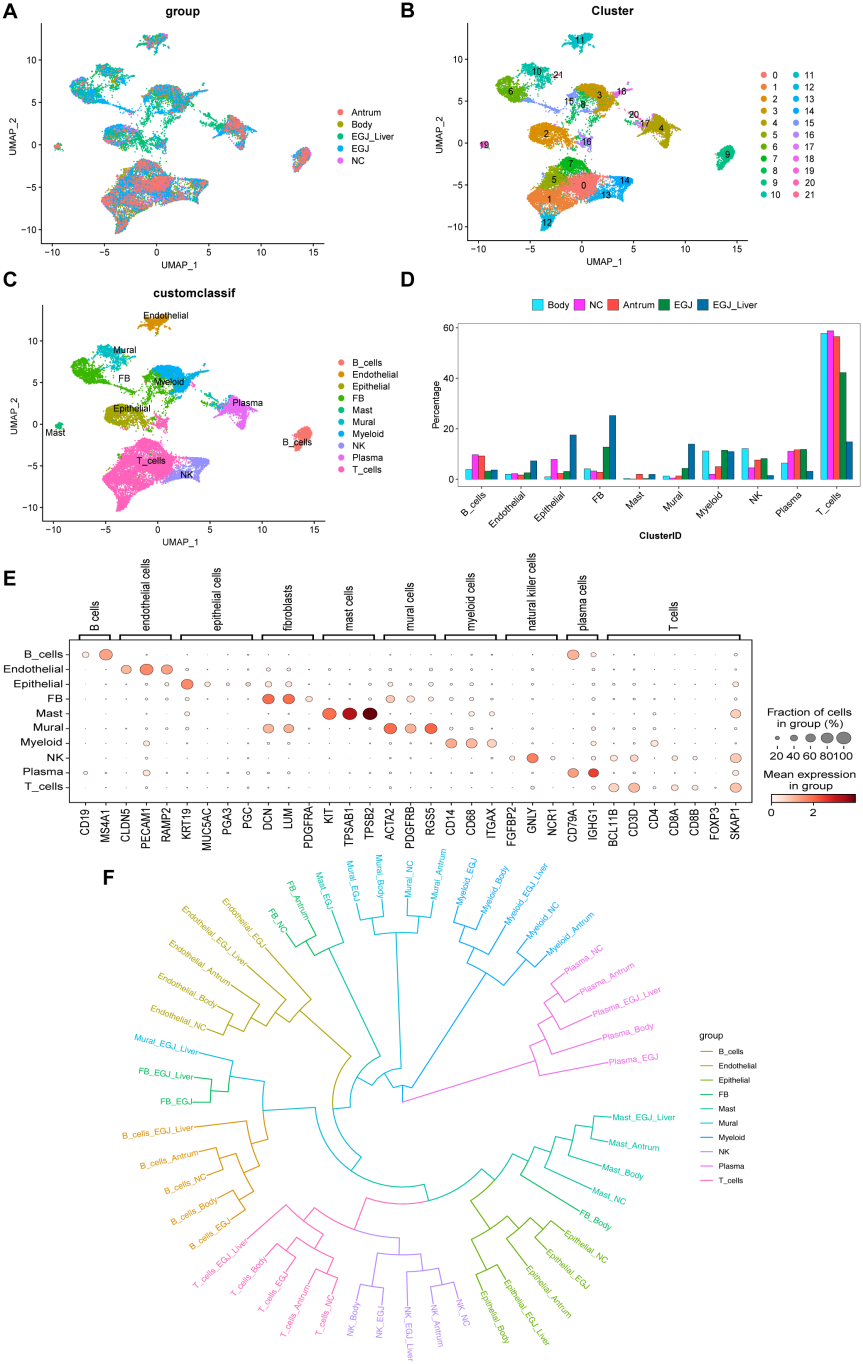
Single-cell RNA sequencing and phylogenetic tree analysis to screen for cell subpopulations associated with AEGJ liver metastasis. **(A)** Uniform manifold approximation and projection (UMAP) plot showing the clustering of different cell subsets in AEGJ liver metastasis (EGJ-liver), AEGJ (EGJ), gastric body cancer (Body), gastric antrum (Antrum) and normal tissues (NC). **(B)** UMAP plot of 14,809 cells from 22 cell clusters. **(C)** Cell clusters were annotated into 10 cell types by marker genes. **(D)** Proportions of 10 cell types across different samples. **(E)** Dot plot showing representative top 3 marker genes for each cell type. **(F)** A phylogenetic tree of major cell types across different samples.

To explore the molecular characteristics and tumor microenvironment of AEGJ liver metastasis, as well as tumor heterogeneity and gene expression differences between AEGJ and gastric cancer in other regions, we performed single-cell RNA sequencing (scRNA-seq) on primary lesions and corresponding normal tissues from two surgically resectable AEGJ cases with liver metastasis. Additionally, we selected a publicly available scRNA-seq dataset (GSE206785) from the GEO database, which includes data from 2 cases of gastric body cancer, 1 case of gastric antrum cancer, and 2 cases of AEGJ for integrated analysis (37). **Figure 1** summarizes the structured workflow of our study. After quality control and merging of self-measured and publicly downloaded data, we obtained a single-cell gene expression profile for 23,426 cells (**Figure 2A**). Uniform Manifold Approximation and Projection (UMAP) was used to reduce the dimensionality of the data, clustering the cells into 22 distinct clusters (**Figure 2B**). Further data correction showed no significant differences in the number of cell clusters across sample sources, suggesting minimal batch effects (**Supplementary Figures S1A, B**).

Next, using t-distributed Stochastic Neighbor Embedding (t-SNE) analysis and expression of typical markers from the Cell Marker database, the 22 cell clusters were annotated into 10 distinct cell types (**Figure 2C**), including: B cells (CD19, MS4A1), Endothelial cells (CLDN5, PECAM1, RAMP2), Epithelial cells (KRT19, MUC5AC, PGA3, PSTAD), Fibroblasts (DCN, LUN, PDGFRA), Mast cells (KIT, TPSB2, TPSAB1), Mural cells (ACTA2, PDGFRB, RGS5), Myeloid cells (CD14, CD68, ITGAX), NK cells (FGFBP2, GNLY, NCR1), Plasma cells (CD79A, IGHG1), T cells (BCL11B, CD3D, CD4, CD8A, CD8B, FOXP3, SKAP1). We analyzed the proportions of each cell type across the samples and found uneven distribution patterns. **Supplementary Figure S1D** shows the distribution of these marker genes. Among the cell types, fibroblasts were more abundant in both the AEGJ and AEGJ liver metastasis groups. Mural cells also had a higher proportion in both groups, with a significantly greater proportion in the AEGJ liver metastasis group compared to the AEGJ group (**Figure 2D**). **Figure 2E** shows the expression levels and distribution of representative marker genes for each cell type and **Supplementary Figure S1C** shows marker genes for each cell clusters. To compare the uniqueness of the main cell lineages across different samples, we constructed a phylogenetic tree (**Figure 2F**). A phylogenetic tree visually represents the developmental trajectories or differentiation routes of cells, derived from genomic sequencing and single-cell analysis. It illustrates the relationships between different cell types, as well as their differentiation and developmental paths. This tree can help analyze similarities and differences in cell differentiation across various tumor types, as well as in different regions or pathological subtypes of the same tumor. By identifying specific subtypes and unique differentiation trends, the phylogenetic tree provides valuable insights into tumor biology (38, 39).

In our analysis, we found that the developmental trajectories and differentiation patterns of fibroblasts differed significantly across AEGJ, gastric body, and gastric antrum. These results suggest that different regions of gastric cancer may harbor unique fibroblast subpopulations. Additionally, when AEGJ undergoes liver metastasis, the developmental trajectories and differentiation routes of its parietal cells diverge notably from those in non-metastatic samples. This finding suggests that parietal cells may play a pivotal role in influencing liver metastasis in AEGJ.

### 3.2 Subpopulations and transcriptome landscape of fibroblast in different samples

**Figure 3:**
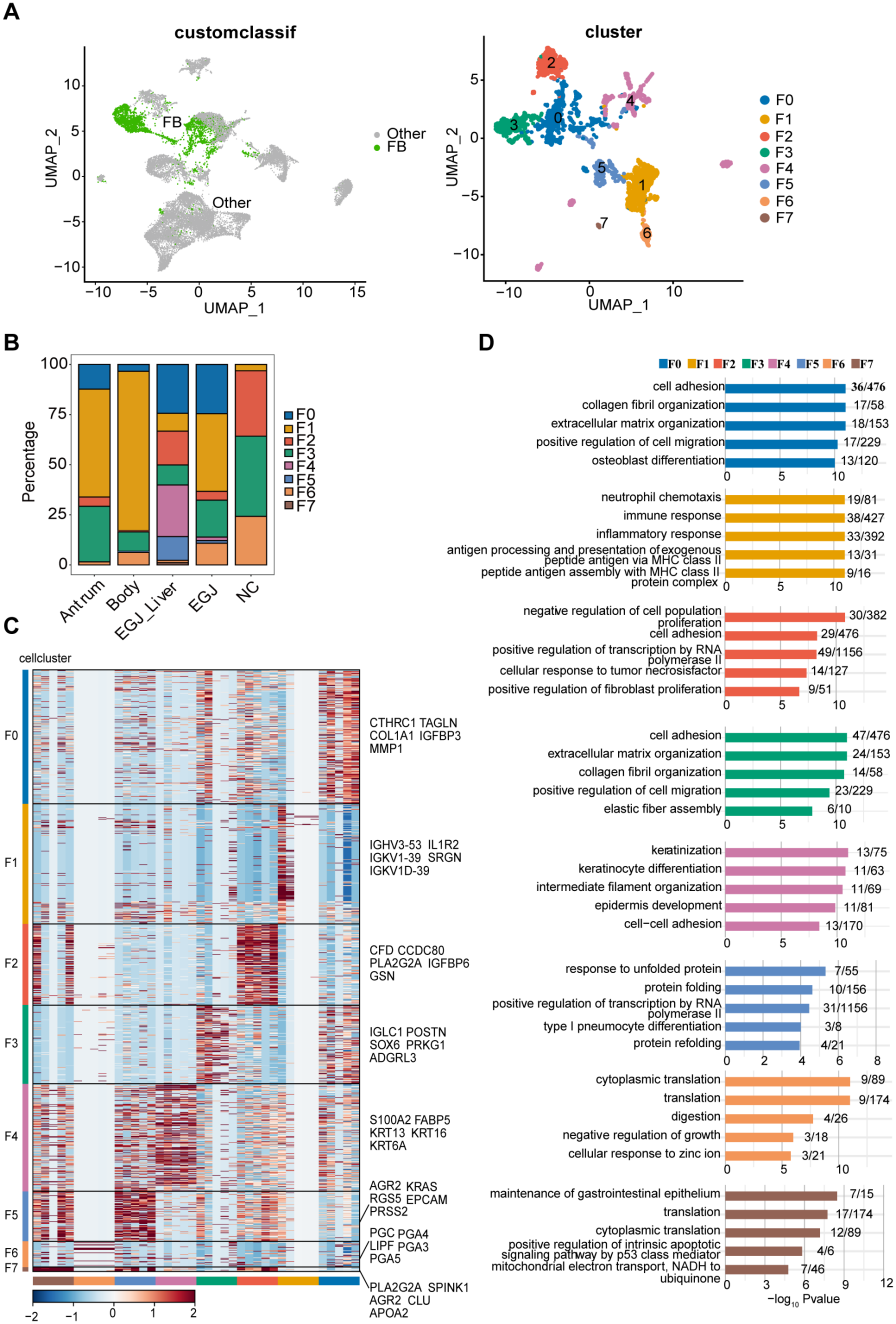
Subpopulations and transcriptome landscape of fibroblast in AEGJ liver metastasis (EGJ-liver), AEGJ (EGJ), gastric body cancer (Body), gastric antrum (Antrum) and normal tissues (NC). **(A)** Uniform manifold approximation and projection (UMAP) plot showing the subtype classification of fibroblast, and it can be divided into 7 clusters. **(B)** Bar charts showing the proportion of each fibroblast subtype in different samples. **(C)** Heatmap showing the top 5 marker genes of each fibroblast subpopulation. **(D)** GO functional enrichment analysis of each fibroblast subpopulation.

Fibroblasts are known for their high plasticity within the tumor microenvironment (40). To investigate whether specific fibroblast subtypes contribute to the liver metastasis process in AEGJ and whether fibroblast heterogeneity influences the molecular differences across gastric cancer sites, we re-clustered the fibroblasts in the analyzed dataset, resulting in 8 distinct clusters (**Figure 3A**). We then examined the distribution of these fibroblast clusters across different samples. Cluster F0 was present in all tumor samples, while F1 was most abundant in gastric body samples, showing a higher proportion than in other STAD regions or normal tissues. Cluster F3 exhibited a higher proportion in gastric antrum and normal tissues compared to other tumor sites. F4 was exclusively found in AEGJ samples, with its proportion significantly elevated in AEGJ liver metastasis compared to non-metastatic AEGJ samples. Clusters F2 and F6 were more abundant in normal tissues than in tumor samples (**Figure 3B**).

We performed clustering analysis based on the expression levels of characteristic genes for each of the 8 fibroblast clusters, highlighting the top five characteristic genes in the figure. Notably, cells within the same cluster exhibited a certain degree of similarity in the expression of these marker genes (**Figure 3C**). Gene ontology (GO) functional enrichment analysis of these characteristic genes revealed distinct biological processes associated with each cluster. F0 and F3 were primarily enriched in processes related to cell adhesion, collagen fiber formation, extracellular matrix organization, and cell migration. F1 was enriched in immune and inflammatory response pathways, while F2 was associated with processes such as inhibition of cell proliferation and RNA polymerase II activity. F4 showed enrichment in biological processes such as cell differentiation, collagen fiber organization, cell development, and cell adhesion (**Figure 3D**).

**Figure 4:**
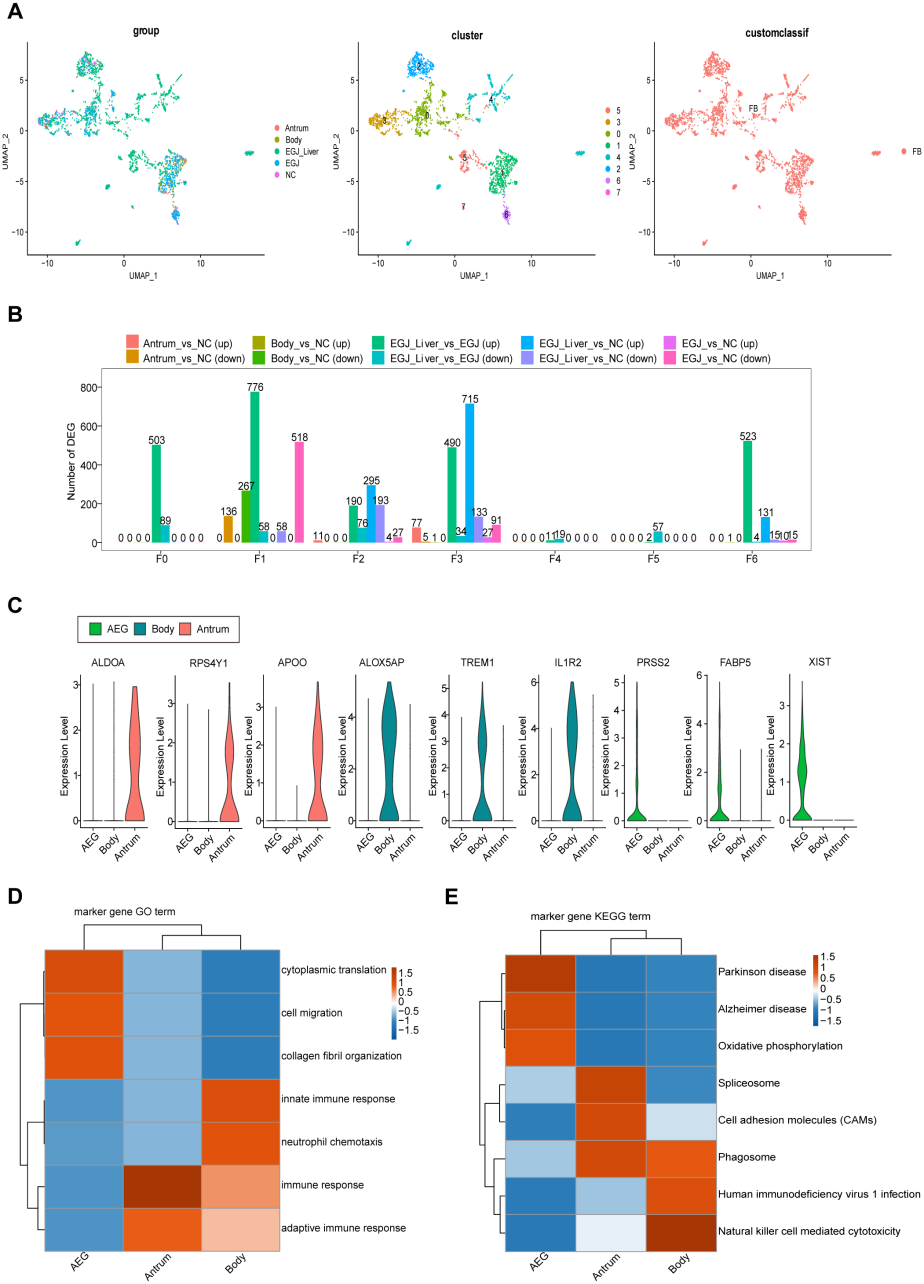
Differences in these characteristic genes in AEGJ and STAD at different sites. **(A)** UMAP plot showing the clustering of different fibroblasts in AEGJ liver metastasis (EGJ-liver), AEGJ (EGJ), gastric body cancer (Body), gastric antrum (Antrum) and normal tissues (NC). **(B)** Differential genes were compared between clusters of fibroblasts in different samples. **(C)** Expression of the three most significantly different genes in AEGJ, body and antrum. **(D, E)** GO and KEGG analysis in different genes from AEGJ, body and antrum.

To examine site-specific differences in characteristic genes in AEGJ and STAD, we conducted differential expression analysis of all fibroblast characteristic genes in tumor and normal tissue groups (**Figure 4A**). We then compared the resulting differentially expressed genes between fibroblast clusters. Characteristic genes in clusters F0, F4, and F5 were significantly differentially expressed only in AEGJ and AEGJ liver metastasis samples, whereas genes in clusters F1, F2, and F3 were significantly differentially expressed across all tumor samples (**Figure 4B**). Our prior phylogenetic tree analysis (**Figure 2B**) revealed distinct developmental trajectories and differentiation patterns of fibroblasts in AEGJ, the gastric body and the gastric antrum, suggesting site-specific fibroblast subpopulations. Thus, we compared the differentially expressed fibroblast characteristic genes across the three tumor types. **Figure 4C** displays the three most significantly differentially expressed genes in each type: FABP5, PRSS2, and XIST in AEGJ; ALDOA, RPS4Y1, and APOO in the gastric antrum; and ALOX5AP, TREM1, and IL1R2 in the gastric body. We conducted functional clustering and pathway enrichment analyses on these differentially expressed characteristic genes. The results revealed that differentially expressed characteristic genes in AEGJ were primarily enriched in pathways related to cell differentiation, migration, collagen fiber organization, and oxidative phosphorylation. In contrast, the gastric antrum and gastric body shared similarities in functional clustering and pathway enrichment, with both enriched in immune response and neutrophil chemotaxis pathways (**Figure 4D**). Additionally, the gastric antrum was enriched in cell adhesion pathways, while the gastric body showed enrichment in NK cell-mediated cytotoxicity pathways. Both the gastric antrum and gastric body were also involved in phagosome-related pathways. Furthermore, the gastric antrum was enriched in spliceosome and cell adhesion molecule (CAM) pathways, whereas the gastric body was enriched in HIV-1 infection and NK cell-mediated cytotoxicity pathways (**Figure 4E**). Integrating these results, we found that the characteristic genes in cluster F4 closely matched the differentially expressed characteristic genes of AEGJ fibroblasts, including FABP5 and XIST. Studies have shown that FABP5 overexpression is associated with poor prognosis in STAD patients. Elevated FABP5 expression in tumor cells promotes gastric cancer cell proliferation and survival, driving tumor progression (41). Fatty acid-binding protein 5 (FABP5) primarily binds and transports long-chain fatty acids, regulating intracellular lipid metabolism. This process is essential for cell membrane construction and energy metabolism (42). FABP5 facilitates rapid gastric cancer cell proliferation by promoting lipid metabolism. Additionally, FABP5 enhances gastric cancer cell migration and invasion by remodeling the cytoskeleton and regulating extracellular matrix degradation (43). X-inactive specific transcript (XIST) is a key long non-coding RNA on the X chromosome, essential for X chromosome inactivation (XCI). Research shows that XIST promotes STAD progression by targeting miR-185 via TGF-β1 (44). Furthermore, XIST regulates STAD progression by acting as a molecular sponge for miR-101, modulating EZH2 expression (45). In summary, the F4 subgroup represents a specific fibroblast subgroup in AEGJ, leading to distinct cell differentiation characteristics and molecular profiles compared to gastric cancers in the gastric antrum and body.

### 3.3 Genes expression changes in fibroblast during AEGJ liver metastasis

**Figure 5:**
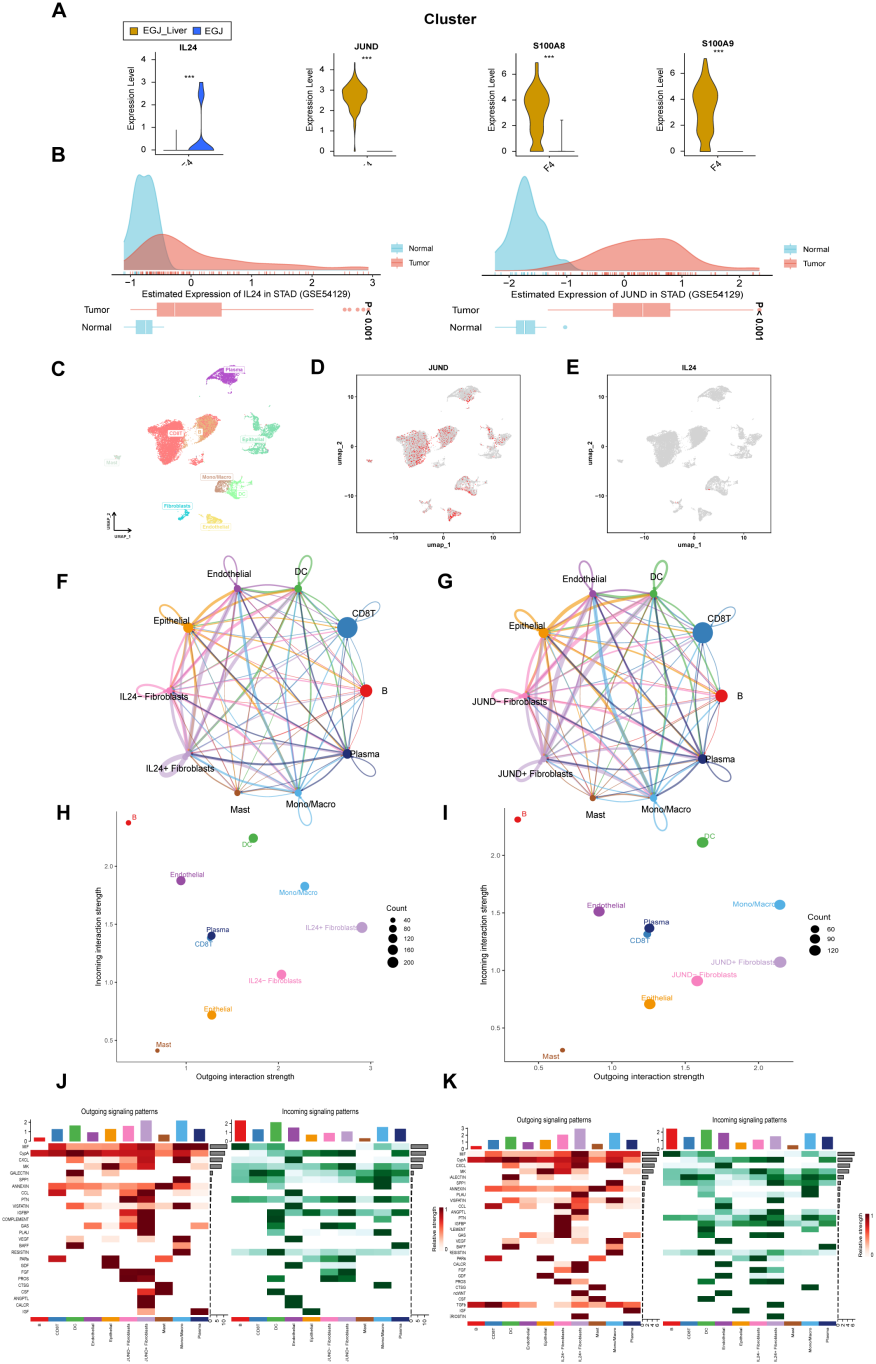
Analysis of significantly genes in F4 fibroblast in AEGJ liver metastasis. **(A)** Violin plot of 4 significantly differentially expressed genes in F4 subtype between AEGJ liver metastasis and AEGJ without liver metastasis. p < 0.05. **(B)** IL24 and JUND genes expression in Stomach adenocarcinoma (STAD) in GEO database (GSE54129). **(C)** UMAP plot showing different cell subsets in public single-cell database STAD-GSE1672972. **(D, E)** The expression and distribution of JUND and IL24 in STAD-GSE1672972. **(F, G)** Circle plot showing the communication strength between interacting cells and JUND/IL24+/- fibroblasts. **(H, I)** Outgoing and incoming interaction strength of the JUND/IL24+/- fibroblasts across cellular groups. **(J, K)** Heatmap showing the overall outward and inward signaling patterns of the JUND/IL24+/- fibroblasts.

F4 represents a specific fibroblast subgroup in AEGJ. Given the observed increase in F4 fibroblasts during AEGJ liver metastasis, we sought to identify the genes driving this expansion. We compared the characteristic genes of F4 fibroblasts in AEGJ liver metastasis samples with those in primary AEGJ samples, identifying four significantly differentially expressed genes (**Figure 5A**).

Among these genes, JUND, S100A8, and S100A9 were highly expressed in AEGJ liver metastasis samples, while IL24 was predominantly expressed in primary AEGJ samples. **Supplementary Figures S2A-D** illustrate the distribution of the four genes within fibroblasts. We validated the expression of these four genes using public GEO data, where all four showed high expression in STAD/AEGJ tumor tissues, aligning with our single-cell sequencing findings (**Figure 5B**, **Supplementary Figures S2E, F**). We further confirmed the expression and distribution of these four genes using a public gastric cancer single-cell database. In the STAD_GSE1672972 database (**Figure 5C**), we observed that IL24 and JUND were enriched in the fibroblast cluster (**Figures 5D, E**). S100A8 and S100A9 were enriched in dendritic cells and macrophages (**Supplementary Figures S2G, H**) and showed no significant expression differences in fibroblasts. The differential expression of IL24 and JUND in fibroblasts within gastric cancer tissues was consistent between our results and the public single-cell data, whereas the results for S100A8 and S100A9 were inconsistent. Using Cell Chat analysis, we mapped the cellular communication landscape, showing interaction affiliations between fibroblasts (with or without JUND/IL24 expression) and other cellular clusters (**Figures 5F, G**). When evaluating interaction intensities for JUND/IL24+/- fibroblasts across cellular groups, we found that dendritic cells showed the strongest combined association (**Figures 5H, I**). Heatmaps of directional signals for the two genes revealed complex interactions among cell populations, with both IL24 and JUND showing significant connections between JUND/IL24+/- fibroblasts and MIF (**Figures 5J, K**).

**Figure 6:**
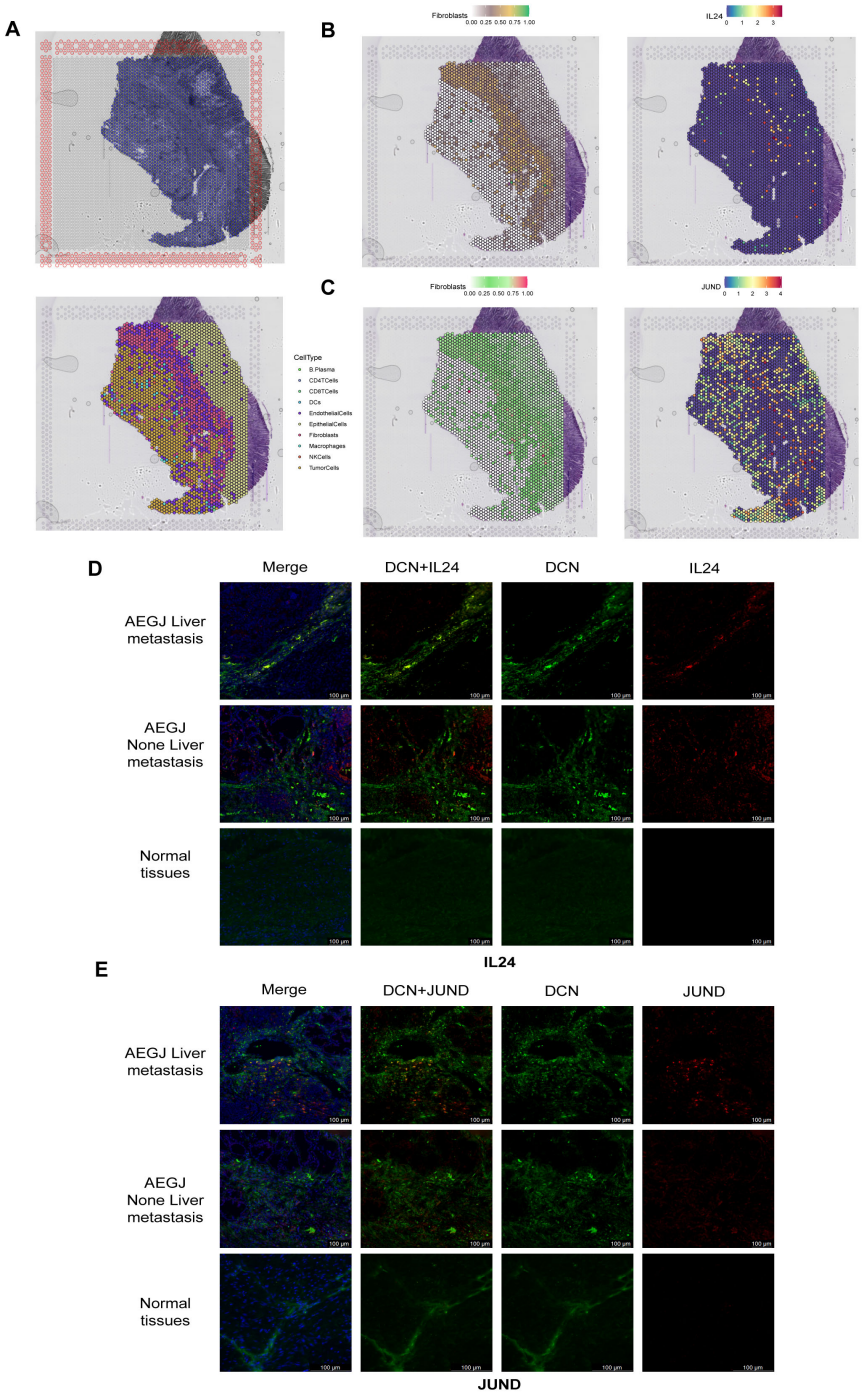
Spatial transcriptome sequencing and immunofluorescence staining validate the significantly gene expression results in F4 fibroblasts. **(A)** Section annotations of hematoxylin-eosin staining (H&E) (up) and spatial images of unsupervised clustering results (down). **(B, C)** The spatial maps show the spatial expression pattern of fibroblasts and marker genes (IL24, JUND) in this study. **(D, E)** Immunofluorescence staining of IL24 and JUND together with DCN (fibroblast) and DAPI (nuclei) (100 µm).

We obtained spatial transcriptomics data for STAD patients from the GEO database’s GSE203612 dataset to verify the expression and distribution of IL24 and JUND (**Figure 6A**). We applied strict quality control measures to the spatial transcriptomics dataset. To accurately assess cell composition at each point on the 10×Visium slide, we used deconvolution analysis, integrating spatial transcriptomics (ST) and single-cell transcriptomics data specific to the cancer type. **Figure 6A** shows the locations of all cell types after spatial transcriptomics deconvolution. **Figures 6B** and **C** reveal that IL24 and JUND expression closely resembles that of fibroblasts, suggesting these genes are primarily expressed by fibroblasts in STAD, consistent with single-cell sequencing results. To further validate our findings, we performed immunofluorescence staining on AEGJ liver metastasis and non-metastatic tissues, using DCN to label fibroblasts in tissue sections, followed by IL24 and JUND staining. Immunofluorescence results showed that IL24 and JUND co-localized with fibroblast markers in AEGJ tumor tissues. IL24 expression was lower in AEGJ liver metastasis than in primary AEGJ, while JUND was highly expressed in AEGJ liver metastasis. This pattern is consistent with our single-cell results, further confirming that IL24 and JUND are closely associated with AEGJ liver metastasis (**Figures 6D, E**).

**Figure 7:**
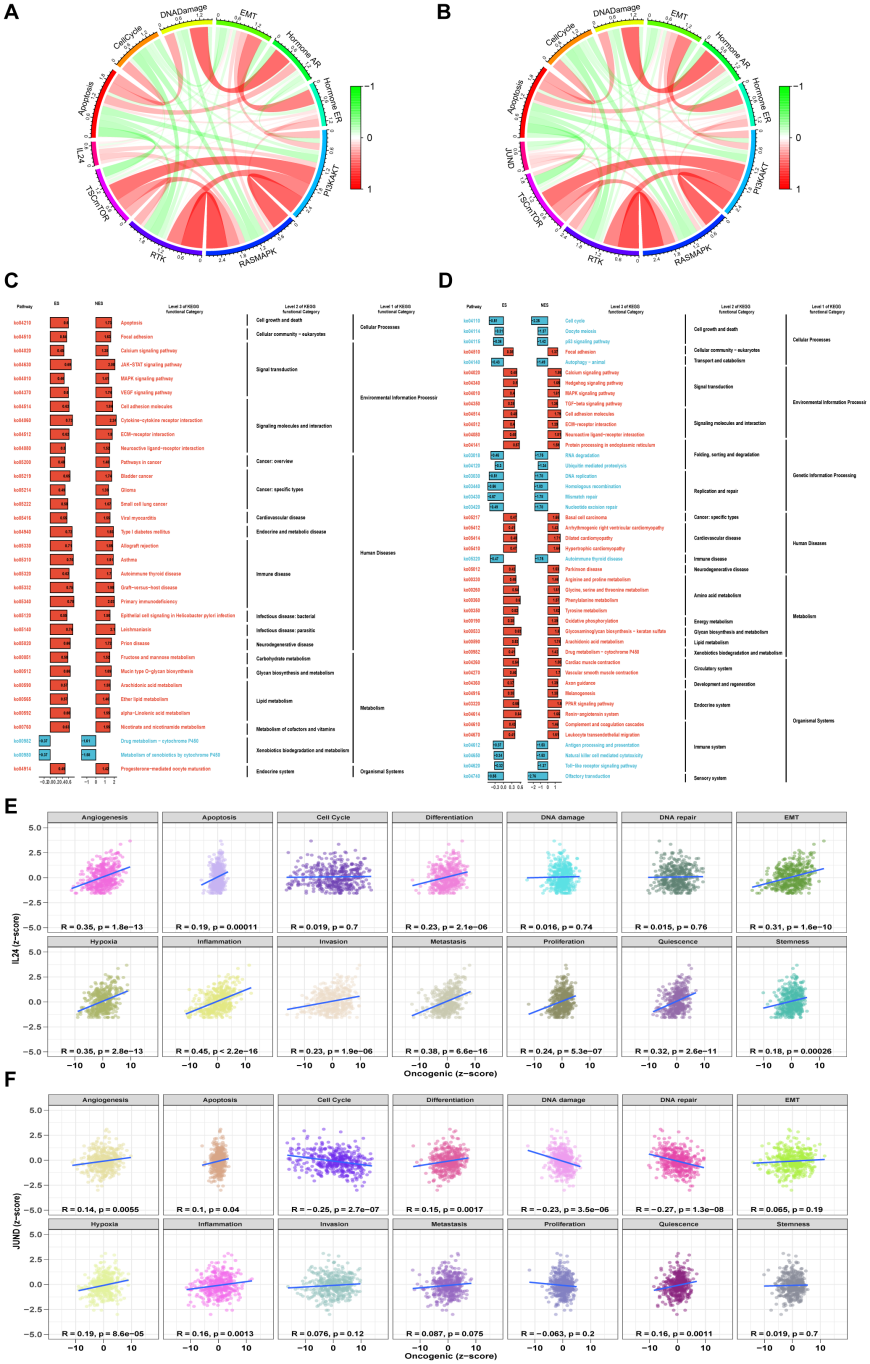
Functional analysis of 2 significantly gene. **(A, B)** Pathway activity scores are calculated for 10 cancer-related pathways (TSC/mTOR, RTK, RAS/MAPK, PI3K/AKT, hormone ER, hormone AR, EMT, DNA damage response, cell cycle, and apoptosis pathways) based on TCPA database in IL24 and JUND. **(C, D)** The pathway enrichment analysis based on the KEGG gene set and the ES of each gene set is calculated, and the ES values of the gene sets are subjected to a significance test and a multiple hypothesis test. Genes with p value less than 0.05 and adjusted p value less than 0.25 are considered significant and are visualized. **(E, F)** Pearson correlation of GSVA scores between z-scores of IL24 and JUND gene expression level and 14 tumor states.

We analyzed the functions of IL24 and JUND. TCPA functional protein analysis showed that in STAD/AEGJ, IL24 is negatively correlated with the DNA damage pathway and positively correlated with the MAPK and PI3K-AKT pathways (**Figures 7A, B**). JUND is positively correlated with the RKT, MAPK, and PI3K-AKT pathways and negatively correlated with the cell cycle. KEGG functional enrichment analysis revealed that high IL24 and JUND expression is enriched in focal adhesion and calcium signaling pathways, suggesting involvement in cell-matrix interactions (**Figures 7C, D**). GSVA analysis suggested that IL24 and JUND expression is significantly positively correlated with the metastasis process in gastric cancer (**Figures 7E, F**). IL24 and JUND interact with the extracellular matrix, affecting fibroblast function and matrix dynamics, thereby influencing AEGJ liver metastasis.

### 3.4 Subpopulations and transcriptome landscape of Mural cell in different samples

**Figure 8:**
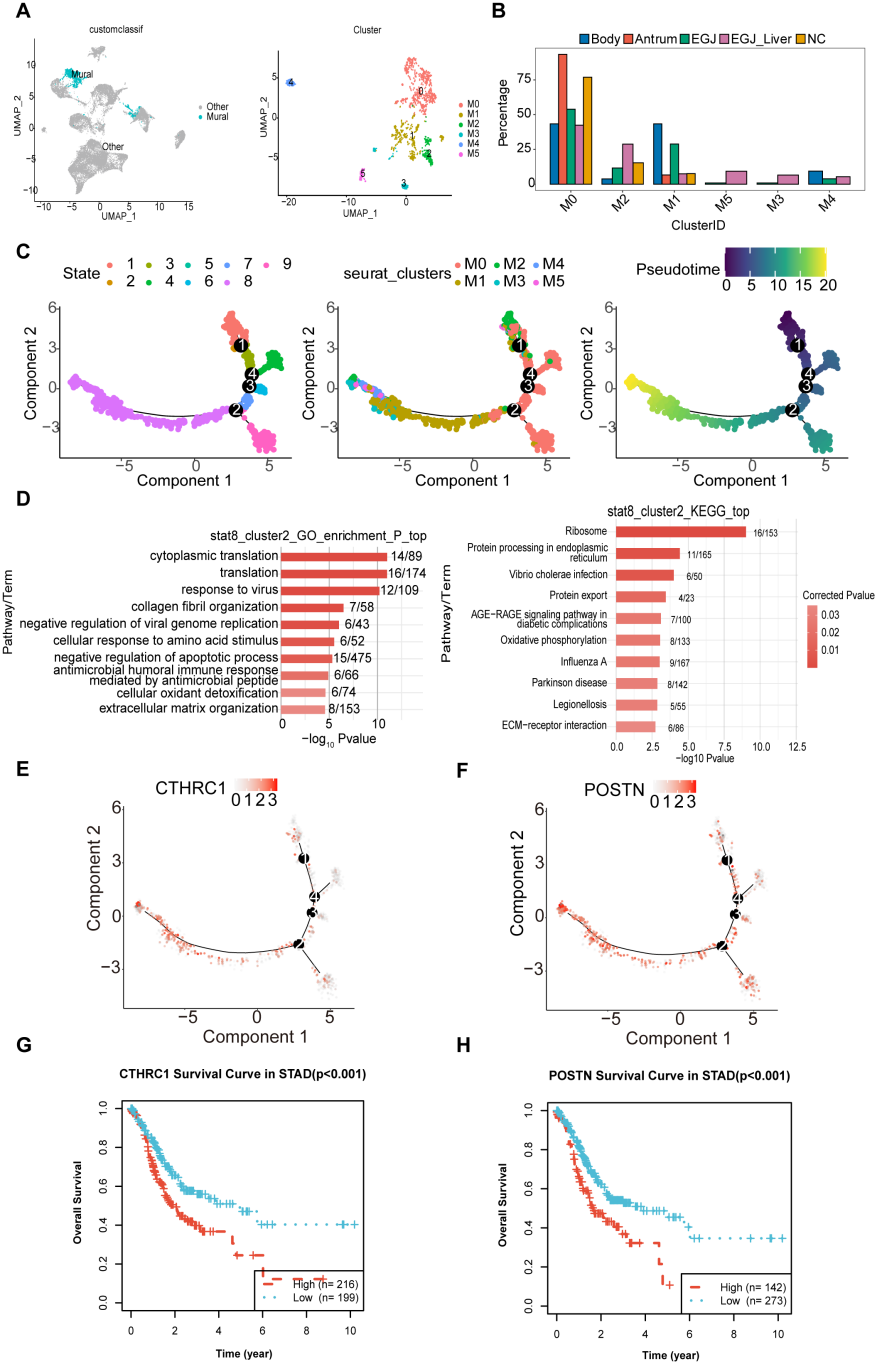
Subpopulations and transcriptome landscape of mural cell in AEGJ liver metastasis (EGJ-liver), AEGJ (EGJ), gastric body cancer (Body), gastric antrum (Antrum) and normal tissues (NC). **(A)** UMAP plot showing the clustering of 6 different mural cells in this study. **(B)** Bar charts showing the proportion of each mural cells subtype in different samples. **(C)** Figure of mural pseudotime, and the color from red (state 1) to purple (state 8) indicates the progression of time. **(D)** The GO and KEGG analysis in endpoints stata8. **(E, F)** 2 cancer-associated fibroblast marker genes, CTHRC1 and POSTN, were clustered in state1 and state8 in pseudotime. **(G, H)** Kaplan-Meier survival analysis for OS (Overall Survival) of CTHRCI and POSTN in STAD.

Phylogenetic tree analysis revealed that the developmental trajectory and differentiation pathway of mural cells differed significantly in AEGJ with liver metastasis compared to non-metastatic samples, suggesting that mural cells may be another cell group influencing AEGJ liver metastasis. Therefore, further analysis of mural cells is required. Mural cells, located on the blood vessel wall, include pericytes and vascular smooth muscle cells (vSMCs). Mural cells play a crucial role in vascular stability and angiogenesis and are an important component of the tumor microenvironment. In this study, we identified 1518 mural cells and clustered them using known markers for pericytes and vascular smooth muscle cells. We identified six clusters (**Figure 8A**). M0 and M1 were present in all samples; M2 was absent in gastric antrum samples; M4 was present only in AEGJ liver metastasis, AEGJ, and gastric body samples; and M3 and M5 were present only in AEGJ liver metastasis and AEGJ samples (**Figure 8B**). Based on annotation, the M0 subgroup comprises pericytes, while the M1, M3, M4, and M5 subgroups comprise vascular smooth muscle cells. We found that marker genes for both pericytes and vascular smooth muscle cells were present in the M2 subgroup, making it unclassifiable as either cell type. This subgroup may represent a mixed cell type undergoing differentiation and is temporarily labeled as “Unknown.” Previous studies report that mural cells can transform into fibroblasts or endothelial cells during disease progression (46).

We conducted pseudotime analysis on mural cell subgroups, identifying 9 cell states and several main branching trajectories (**Figure 8C**). Pseudotime analysis preliminarily identified state 1 as the starting point of mural cell development and state 8 as the potential endpoint. Analyzing pseudotime results by mural cell classification revealed distinct pseudotime directions for pericytes and vascular smooth muscle cells, with a clear differentiation trajectory for the M2 subgroup. The M2 subgroup appeared at both the starting and ending points of pseudotime, suggesting that this cluster represents differentiating mural cells. To further investigate the differentiation direction of the M2 subgroup, we analyzed its differentiation endpoints. We first extracted M2 cells from pseudotime endpoint state 8 for GO and KEGG enrichment analysis, which showed GO enrichment in collagen fiber organization (**Figure 8D**). KEGG analysis indicated enrichment in protein processing and transport processes. These enrichment results suggest that the pseudotime endpoint of the M2 subgroup may be fibroblasts. We further analyzed differentially expressed genes in the M2 subgroup in state 8 to identify fibroblast-related genes. Two cancer-associated fibroblast marker genes, CTHRC1 and POSTN, were clustered here (**Figures 8E, F**). Previous studies indicate that fibroblasts with high CTHRC1 and POSTN expression are markers for gastric cancer-associated fibroblasts (24, 37). KM survival analysis shows that patients with high expression of these genes have poorer survival in STAD (**Figures 8G, H**). This suggests that the developmental trajectory from state 1 to state 8 of the M2 subgroup may represent the transformation of mural cells into gastric cancer-associated fibroblasts. Changes in the tumor microenvironment due to this differentiation process may contribute to AEGJ progression or metastasis.

### 3.5 SFRP2+ pericytes participate in the process of AEGJ liver metastasis

**Figure 9:**
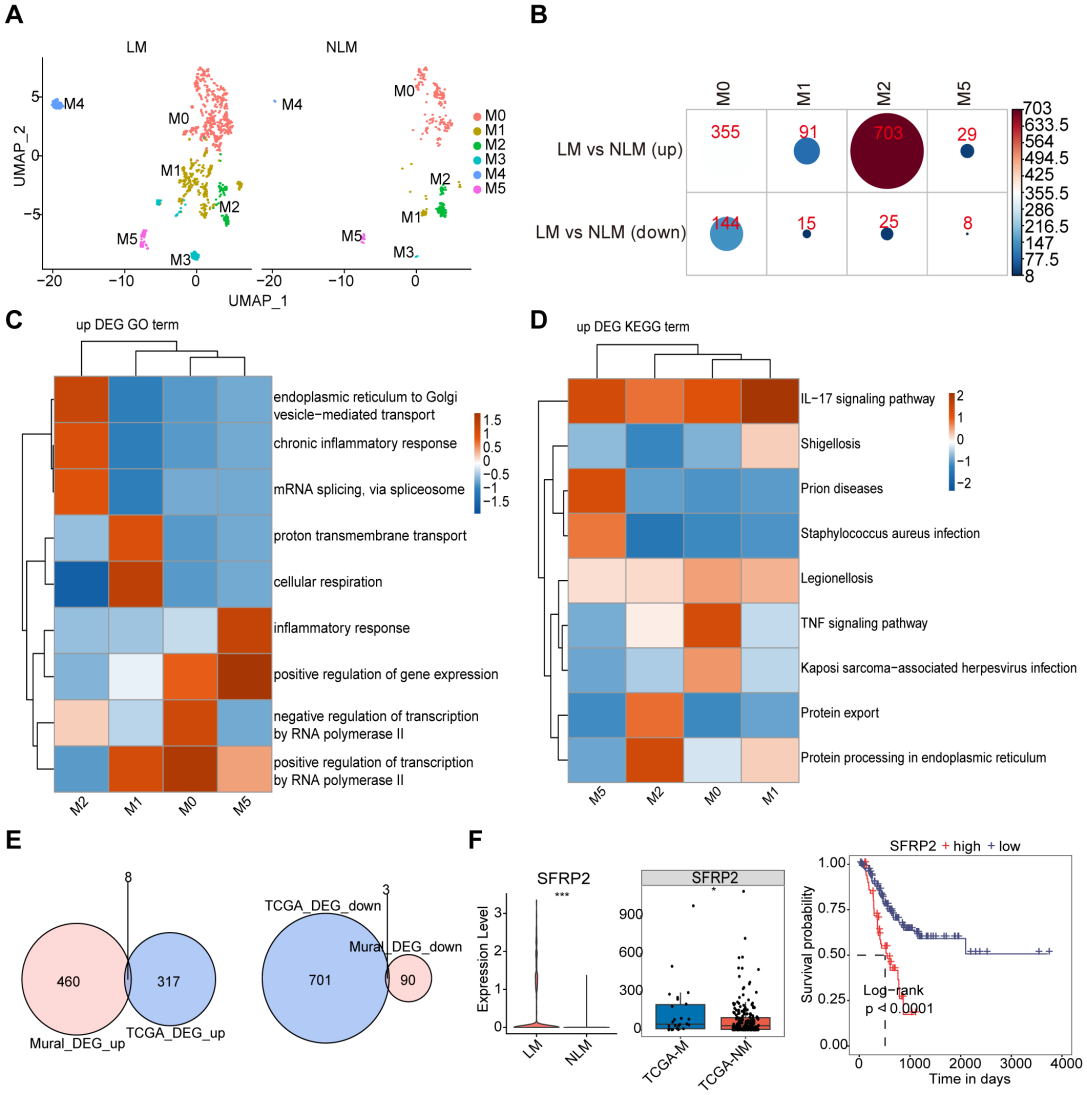
mural cell marker genes associated with AEGJ liver metastasis. **(A)** UMAP analysis compared mural cells between AEGJ liver metastasis and non-metastasis groups. **(B)** The differentially expressed genes between AEGJ liver metastasis and non-metastasis groups, with results showing that differentially expressed genes mainly appeared in M0, M1, M2, and M5, with the highest number in the M2 subgroup. **(C, D)** GO and KEGG functional clustering analysis of all differentially expressed genes. **(E)** Venn plot show the DEGs (differentially expressed genes) between our study and TCGA-STAD. **(F)** SFRP2 expression was higher in AEGJ liver metastasis than in the non-metastasis group and higher in the TCGA-STAD distant metastasis group than in the non-metastasis group and survival analysis indicated that patients with high SFRP2 expression had poorer prognosis.

To further identify mural cell marker genes associated with AEGJ liver metastasis, we compared mural cells between AEGJ liver metastasis and non-metastasis groups, showing subgroup distributions in **Figure 9A**. We compared the differentially expressed genes between the two groups, finding that they were mainly concentrated in M0, M1, M2, and M5, with the highest number in the M2 subgroup. The number of upregulated differentially expressed genes was significantly higher than that of downregulated genes (**Figure 9B**). GO and KEGG analysis of all differentially expressed genes from the comparison revealed that upregulated genes were commonly enriched in the IL-17 signaling pathway. M0 was enriched in gene expression regulation and the TNF signaling pathway, M1 in protein transport, M2 in protein transport and chronic inflammation, and M5 in the inflammatory response (**Figures 9C, D**). To further analyze these differentially expressed genes (DEGs), we screened TCGA-STAD database transcriptomic data on distant metastasis, identified DEGs, and intersected them with group DEGs, yielding 11 DEGs (**Figure 9E**). These included 8 upregulated and 3 downregulated genes. Survival analysis of these 11 genes identified 6 significantly associated with prognosis: CALD1, HSPH1, MAL2, MYL9, SFRP2, and TAGLN (**Supplementary Figure S3**). Of these 6 genes, only SFRP2’s survival results were consistent with its expression patterns. SFRP2 expression was higher in AEGJ liver metastasis than in the non-metastasis group, and also higher in the TCGA-STAD distant metastasis group compared to the non-metastasis group. Survival analysis indicated that patients with high SFRP2 expression had a poorer prognosis (**Figure 9F**).

**Figure 10:**
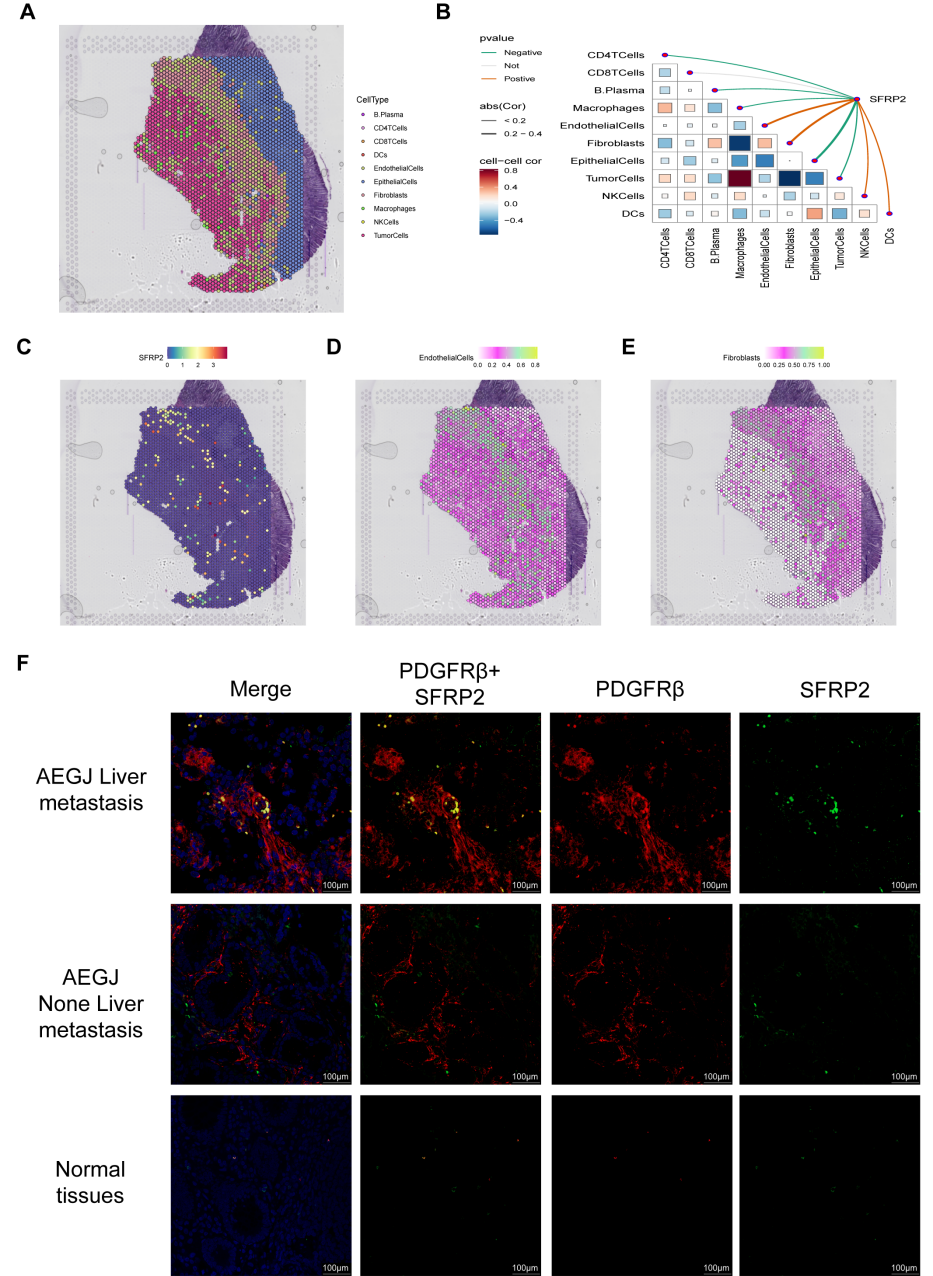
The expression analysis of SFRP2 in STAD. **(A)** Localization of all cells after spatial transcriptome deconvolution. **(B)** Spearman map of gene expression and microenvironment components at spatial transcriptome resolution. **(C)** Spatial transcriptome mapping of single gene SFRP2. **(D, E)** Spatial mapping of SFRP2 in fibroblast and endothelial cell partitions. **(F)** Immunofluorescence staining of SFRP2 expression together with PDGFR-β (mural–pericyte) and DAPI (nuclei) (100 µm), the result show SFRP2 higher expression in AEGJ liver metastasis and lower expression in AEGJ without liver metastasis.

To further validate SFRP2 expression and distribution in STAD, we analyzed spatial transcriptome data (**Figure 10A**). We used Spearman’s correlation analysis to calculate correlations between cellular contents in all spatial transcriptomic spots, as well as between cellular contents and gene expression levels, and visualized the results with the linkET package (**Figure 10B**). The results showed that SFRP2 expression was significantly and positively correlated with fibroblast and endothelial cell content in the spots, which was further verified by the single-gene spatial transcriptome localization map (**Figures 10C–E**). Combined with previous single-cell sequencing results, we further confirmed that SFRP2 is involved in genes related to the STAD tumor microenvironment and may participate in vascular-related tumor functions or processes. Since mural cells are composed of pericytes and vascular smooth muscle cells, to further determine the subcellular localization of the SFRP2 gene in mural cells, we used tissue immunofluorescence to detect SFRP2 expression in AEGJ liver metastasis, AEGJ, and normal tissues, and identified the expression localization of the SFRP2 gene in pericytes (**Figure 10F**). The results showed that SFRP2 was abundantly present in pericytes of patients in the AEGJ liver metastasis group, significantly higher than in the non-metastasis group and normal group, indicating that SFRP2+pericytes significantly influence AEGJ liver metastasis.

**Figure 11:**
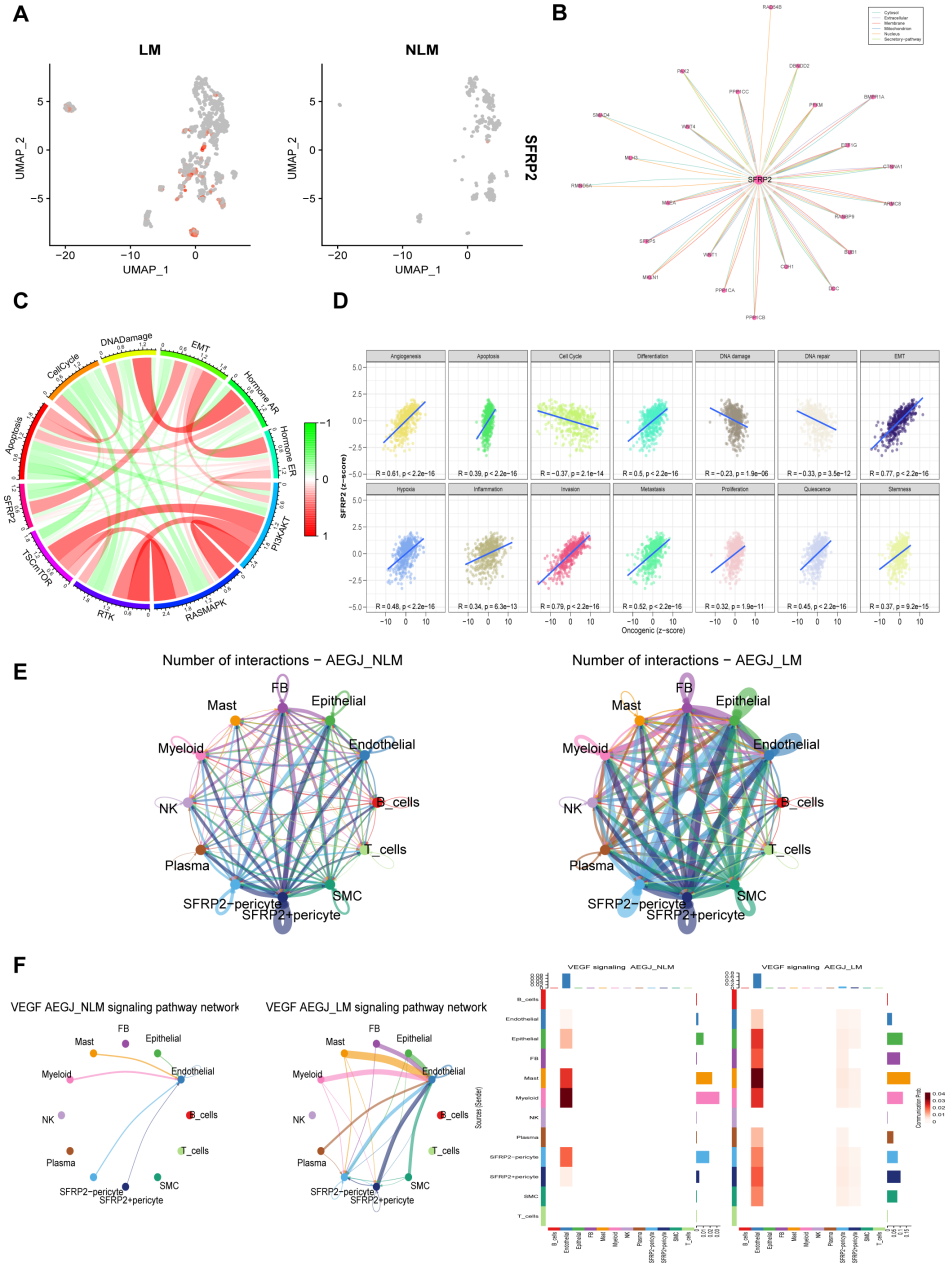
The functional analysis of SFRP2 in AEGJ liver metastasis. **(A)** UMAP plot show the different expression of SFRP2 gene between AEGJ liver metastasis and none metastasis. **(B)** Protein-protein interaction result in SFRP2 in the comPPI database. **(C)** Correlation between SFRP2 gene expression and pathway level quantification of functional proteins by TCPA-RPPA sequencing. **(D)** GSVA activity analysis suggests that the SFRP2 gene is significantly positively correlated with the metastasis process in STAD/AEGJ. **(E)** Circle plot showing the different communication strength between interacting cells and SFRP2+/- pericyte in AEGJ liver metastasis and AEGJ none liver metastasis. **(F)** Intercellular signaling pathway Chord diagrams and heatmap between SFRP2+/- pericyte and VEGF in AEGJ liver metastasis and non-liver metastasis groups.

We further analyzed the distribution of the SFRP2 gene among different pericyte subgroups (**Figure 11A**). We speculate that the SFRP2 gene may serve as a potential molecular marker for AEGJ liver metastasis. comPPI protein interaction network suggests that the SFRP2 gene is associated with WNT proteins (**Figure 11B**). TCPA functional protein analysis indicates that in STAD/AEGJ, SFRP2 is significantly positively correlated with epithelial-mesenchymal transition (EMT) and negatively correlated with the cell cycle (**Figure 11C**). GSVA activity analysis suggests that the SFRP2 gene is significantly positively correlated with the metastasis process in STAD/AEGJ (**Figure 11D**). We found that both the intensity and density of cellular communication involving SFRP2+ pericytes were significantly higher in the AEGJ liver metastasis group than in the non-liver metastasis group (**Figure 11E**). In the AEGJ liver metastasis group, SFRP2+ pericytes were closely associated with the angiogenesis-related VEGF pathway (**Figure 11F**), suggesting that SFRP2+ pericytes may play a role in tumor angiogenesis-related pathways and mechanisms. Those further indicate the relevance and potential role of SFRP2 in AEGJ tumor metastasis.

## 4 Discussion

Adenocarcinoma of the esophagogastric junction (AEGJ) is a highly aggressive malignant tumor of the digestive tract with a poor prognosis and an increasing incidence worldwide. Liver metastasis is a common distant metastasis in AEGJ, significantly impacting patient survival rates. The mechanisms underlying AEGJ liver metastasis remain unclear, and in-depth research into these molecular pathways is essential for improving patient prognosis. In this study, we conducted the first single-cell transcriptome analysis of primary and adjacent normal tissues of AEGJ with liver metastasis, integrating these data with public AEGJ single-cell transcriptome datasets. This analysis identified key components of the tumor microenvironment (TME) involved in AEGJ liver metastasis and revealed specific cell subpopulations and molecules associated with cancer cell extravasation. These findings advance our understanding of AEGJ liver metastasis mechanisms and suggest new therapeutic targets for AEGJ.

Why is AEGJ prone to hematogenous metastasis? Anatomically, AEGJ spans both the thoracic and abdominal regions, with dual blood return pathways from each cavity. Its proximity to the liver’s blood supply further increases the risk of liver metastasis (47–49). However, AEGJ liver metastasis is a complex process that cannot be fully understood or inhibited from an anatomical perspective alone; it requires a deeper, molecular-level analysis. In brief, cancer cells first penetrate the basement membrane and extracellular matrix (ECM) around the primary tumor, then enter the bloodstream (hematogenous metastasis) or lymphatic system (lymphatic metastasis). This process is called intravasation (50). In the circulatory system, cancer cells interact with vascular endothelial and wall cells, crossing vessel walls to enter new tissues—a process called extravasation. In their new location, cancer cells must adapt to the local microenvironment and begin to proliferate. This process involves the tumor microenvironment, including immune cells, fibroblasts, and the extracellular matrix. Cancer cells may induce angiogenesis to secure essential nutrients and oxygen, promoting tumor growth while evading immune surveillance and clearance in their new environment (51, 52). Cancer growth in a new location often starts as a tiny, undetectable stage known as micrometastasis. Over time, these micrometastases gradually develop into clinically detectable tumors, called metastatic foci. This microscopic process involves biological mechanisms such as epithelial-mesenchymal transition, intravasation, extravasation, immune evasion, and multiple signaling pathways and molecules, including growth factors, chemokines, and cell adhesion molecules. These factors regulate the growth, survival, migration, and invasion of cancer cells. Key pathways in cancer metastasis include epidermal growth factor receptor (EGFR), Wnt/β-catenin, and transforming growth factor-β (TGF-β) (53, 54).

Currently, single-cell sequencing technology enables the analysis of various microscopic biological processes involved in tumor metastasis, aiding in identifying key targets and offering new therapeutic insights (55–57). Single-cell sequencing can analyze various stages of tumor metastasis, identify malignant cell subpopulations with metastatic potential, or detect cancer stem cell populations influencing metastasis direction. Jiang et al. used single-cell sequencing to analyze liver, peritoneal, lymphatic, and ovarian metastasis samples from six gastric cancer patients, identifying highly invasive malignant cell subpopulations. This malignant epithelial subgroup exhibits invasive characteristics, a tendency for intra-abdominal metastasis, and a tumor stem cell phenotype induced by epithelial-mesenchymal transition. Furthermore, a 20-gene signature of lymph node-derived exhausted CD8+ T cells was identified to predict lymph node metastasis (58). Single-cell sequencing can also analyze the tumor microenvironment promoting metastasis, including endothelial cells, fibroblasts, and the extracellular matrix. Wang et al. conducted single-cell sequencing on six colorectal cancer (CRC) liver metastasis patients, mapping the cellular landscape of CRC and matched liver metastases. They identified cancer-associated fibroblasts linked to CRC liver metastasis and described the associated tumor microenvironment (59). Single-cell sequencing can also analyze immune cell subpopulations associated with tumor metastasis, exploring how metastatic cancer evades immune surveillance, including analyses of T cells, B cells, and macrophages. Elham Karimi et al. used single-cell sequencing to analyze the immune landscape of 139 high-grade glioma and 46 brain metastasis patients, identifying immune resistance mechanisms related to brain metastasis and differences in immune landscapes between primary tumors and brain metastases (60). In this study, we used single-cell sequencing to identify an abnormally expanded fibroblast subgroup in AEGJ liver metastasis, potentially related to IL24 and JUND gene overexpression. This subgroup increase may alter the tumor microenvironment in AEGJ, potentially promoting liver metastasis. The tumor microenvironment (TME) plays a crucial role in tumor metastasis. The TME comprises cancer cells, stromal cells, immune cells, vascular endothelial cells, and various extracellular matrix (ECM) components. Interactions among these components influence tumor growth, invasion, and metastasis. Fibroblasts, particularly cancer-associated fibroblasts (CAFs), are crucial components of the TME that significantly influence tumor progression and metastasis. Kalluri discussed CAFs origin and activation, detailing how these cells undergo phenotypic changes in response to tumor-derived signals (61). This activation process involves complex signaling pathways, including TGF-β and Wnt, which are crucial for CAF-mediated enhancement of cancer cell invasion and metastasis. The study also highlighted therapeutic strategies aimed at reprogramming or inhibiting CAFs activity to mitigate their pro-metastatic effects. Sahai et al. explored bidirectional communication between CAFs and the immune microenvironment, showing that CAFs can modulate immune cell recruitment and function within the TME. Their findings suggest that CAFs establish an immunosuppressive environment that promotes tumor progression and metastasis, offering insights for developing therapies targeting both CAFs and immune components (62).

Recent studies have shed light on the diverse roles of CAFs in promoting gastric cancer metastasis. For example, Roges et al. found that CAFs modulate the Wnt/PCP signaling pathway in gastric cancer cells via the cytokine ROR2, promoting their migration and metastasis. The Wnt/PCP co-receptor ROR2 can be directly transferred from CAFs to gastric cancer cells, triggering JNK signaling, actin polarization, and directional migration in these cells (63). Zhang et al. also found that the HAPLN1 gene is markedly upregulated in gastric cancer CAFs. Gastric cancer cells activate fibroblasts via the TGF-β1/Smad2/3 signaling pathway, enhancing HAPLN1 expression to promote tumor migration and invasion. Elevated HAPLN1 expression in CAFs enhances gastric cancer invasion and metastasis via ECM remodeling (64). CAFs-gastric cancer cell interactions confer greater metastatic potential on gastric cancer cells, leading to metastasis to other sites. Lu et al. analyzed fibroblast secretory characteristics in gastric cancer metastasis, finding that CAFs secretion play a crucial role in shaping the pre-metastatic tumor microenvironment. SLIT2, an axon guidance protein produced by CAFs, promotes metastasis of gastric cancer cell lines AGS and MKN45 by binding to the roundabout guidance receptor 1 (ROBO1) (65). CAFs can also interact with gastric cancer cells through adhesion pathways, inducing their metastasis. Zhang et al. found that CPNE8 promotes STAD progression by regulating focal adhesion. This effect can be reversed by the FAK inhibitor GSK2256098 or by FAK knockout. Additionally, CPNE8 is strongly associated with tumor-associated fibroblast and immune cell infiltration, with high expression predicting poor outcomes for immune checkpoint therapy in gastric cancer (66).

Our study found that AEGJ liver metastasis is associated with high JUND expression and low IL24 expression in liver metastatic fibroblasts. This correlation may involve interaction pathways between gastric cancer cells and fibroblasts, such as focal adhesion and calcium signaling. Activator Protein-1 (AP-1) is a transcription factor composed of proteins from the JUN, FOS, ATF, and MAF families. These proteins form homodimers or heterodimers that bind specific DNA sequences to regulate gene expression. The JUN family includes c-Jun, JunB, and JunD; c-Jun, one of the most studied AP-1 proteins, is involved in cell proliferation, differentiation, and apoptosis. The FOS family includes c-Fos, FosB, Fra-1, and Fra-2. c-Fos often acts with c-Jun, participating in cell proliferation and survival. AP-1 regulates the expression of genes involved in key cellular processes, including cell proliferation, apoptosis, differentiation, and immune response. In cancer, AP-1 is a key regulator of oncogenic processes, including tumor growth, metastasis, and therapeutic targeting (67).

Studies have shown that AP-1 promotes STAD progression by upregulating oncogenes and influencing the tumor microenvironment. AP-1 modulates cytokine expression and other factors that shape the tumor microenvironment, promoting inflammation and immune evasion. Mitsuno et al. found that Helicobacter pylori induced AP-1 activation and expression in gastric cancer cells through the ERK signaling pathway. Helicobacter pylori infection in the gastric mucosa induces IL-8, IL-6, and TNF-α, with AP-1 binding sites in their promoter regions. This activation subsequently triggers intracellular signaling, leading to inflammation and immune responses (68). Another study explored the mechanisms of hyperproliferation in Helicobacter pylori infected gastric epithelial cells, focusing on NF-κB and AP-1 activation. They found that this activation increases β-catenin and c-Myc expression, key regulators of cell proliferation. AP-1 specifically contributes to gastric cancer development by upregulating these oncogenic factors, promoting the hyperproliferative state linked to Helicobacter pylori infection (69). Therefore, AP-1 is considered crucial in Helicobacter pylori-induced STAD, as it induces inflammation and immune responses in normal gastric mucosal cells. Shi et al. investigated AP-1 (JUND) in STAD, showing that catecholamines upregulate MMP-7 expression, promoting tumor invasion and metastasis through AP-1 activation. Their findings reveal the pathway linking stress-related hormones to enhanced STAD progression through AP-1 activation (70).

AP-1 serves as an important initiating factor in gastric cancer development and is crucial for its progression and metastasis. AP-1 activation increases c-Myc and β-catenin expression, both essential for cell proliferation and survival in gastric cancer. Huang et al. found that IL-1β-induced p38 activation activates the AP-1 binding site in the MMP9 promoter. This activation is significantly associated with lymph node metastasis and extramural invasion. AP-1 facilitates tumor invasion and metastasis by regulating matrix metalloproteinases (MMPs) and other factors (71).

IL24, part of the IL-10 cytokine family, is recognized as a tumor suppressor with significant anti-cancer properties. IL24 is also referred to as melanoma differentiation-associated gene 7 (mda-7). IL24 is involved in various biological processes, primarily known for its roles in immune response and cancer biology. IL24 selectively induces apoptosis in cancer cells, sparing normal cells, making it an appealing therapeutic candidate (72). IL24 selectively induces apoptosis in a variety of cancer cell types, sparing normal cells (73). This selective apoptosis is mediated by multiple pathways, including JAK/STAT, p38 MAPK, and ERK (74). IL24 modulates immune response by enhancing immune cell activity against cancer cells. IL24 promotes secretion of cytokines that activate immune responses against tumors (75). Additionally, IL24 increases cancer cell sensitivity to chemotherapy and radiation, enhancing their effectiveness (76). Studies indicate that IL24 expression is often downregulated in STAD due to promoter hypermethylation. Restoring IL24 expression can inhibit gastric cancer cell growth and proliferation. IL24 induces apoptosis in gastric cancer cells by activating apoptotic pathways and upregulating pro-apoptotic proteins like Bax and caspase-3 (77). IL24 inhibits STAD metastatic potential by reducing invasive capabilities. This occurs partly through extracellular matrix modulation and inhibition of enzymes like MMPs that facilitate invasion (78).

The formation of new blood vessels in tumor tissues and the extravasation of cancer cells through vessel walls into other parts of the body are critical factors in tumor metastasis. Mural cells, which form blood vessels, play a crucial role in this process. In our study, we identified a mural cell subpopulation linked to AEGJ liver metastasis, and pseudotime analysis indicates that its differentiation into cancer-associated fibroblasts is a crucial step in AEGJ liver metastasis. In AEGJ, high SFRP2 expression in pericytes, a component of mural cells, is a key marker for liver metastasis. This may be linked to enhanced pericyte-mediated extravasation and the stimulation of epithelial-mesenchymal transition in tumor cells. Mural cells, including pericytes and vascular smooth muscle cells (VSMCs), play a vital role in maintaining vascular stability and function (79). Pericytes cover the outer surface of capillaries and venules, regulating vascular formation, maturation, and homeostasis through direct contact with endothelial cells and by secreting signaling molecules (80).

Pericytes stabilize and mature newly formed blood vessels by producing extracellular matrix components and secreting growth factors like VEGF and PDGF. This stabilization is crucial for maintaining the blood supply needed for tumor growth. However, in the tumor microenvironment, pericyte coverage may be irregular, leading to aberrant angiogenesis characterized by leaky and dysfunctional blood vessels (81). These abnormal vessels not only support tumor growth but also create a chaotic microenvironment that promotes cancer cell invasion and metastasis (82). Pericytes play a critical role in metastasis by regulating tumor vasculature and the extracellular matrix. They produce MMPs and other enzymes that degrade the extracellular matrix, thereby facilitating tumor cell invasion (83). Additionally, pericytes contribute to forming a supportive niche for cancer stem cells, which are essential for tumor initiation, progression, and resistance to therapy (84). The ability of pericytes to remodel the matrix and interact with cancer stem cells highlights their importance in the metastatic cascade (85).

Secreted Frizzled-Related Protein 2 (SFRP2) is a key regulator of the Wnt signaling pathway. It binds to Wnt proteins, blocking their interaction with Frizzled receptors and inhibiting the activation of the Wnt signaling pathway. The Wnt signaling pathway plays a crucial role in cell proliferation, differentiation, and migration. Its abnormal activation is strongly linked to the occurrence and progression of various cancers (86). SFRP2 acts as a tumor suppressor, and many studies have shown that SFRP2 downregulation occurs due to promoter hypermethylation in several types of cancer (87). HYo et al. found that in STAD, LUCAT1 epigenetically downregulates the tumor suppressor gene SFRP2, regulating the activation of the Wnt/β-catenin signaling pathway and promoting the proliferation and differentiation of gastric cancer cells (88, 89). SFRP2 expression appears to be involved in tumor aggressiveness and invasiveness, as indicated by the most significant SFRP2 downregulation in aggressive and invasive pituitary adenomas compared to less aggressive or invasive tumor types (90, 91).

Conversely, the over expression of SFRP2 in cancer cell lines and tumor tissues has also been described. | Conversely, overexpression of SFRP2 in cancer cell lines and tumor tissues has also been reported. High expression of SFRP2 has been observed in osteosarcoma cells, where its upregulation promotes cell proliferation and migration, endowing them with metastatic potential (92). In colorectal cancer, upregulation of the SOX2 gene in colonic stromal cells induces SFRP2 overexpression in cancer-associated fibroblasts, thereby promoting tumor formation (93).

The formation of the tumor vasculature is a critical process in tumor progression and metastasis. Newly formed blood vessels in the tumor microenvironment provide a pathway for cancer cell spread and subsequent metastasis (94). It has been established that microvascular density within tumors correlates with their metastatic potential (95). The Wnt signaling pathway promotes tumor angiogenesis and endothelial cell survival (96). Elevated levels of active β-catenin in tumor cells lead to overexpression of vascular endothelial growth factor (VEGF), a key pro-angiogenic factor that stimulates blood vessel formation (97). Furthermore, matrix metalloproteinases (MMPs) are upregulated through the canonical Wnt signaling pathway, facilitating extracellular matrix remodeling during angiogenesis (98). The β-catenin-independent Wnt/Ca2+ pathways are also implicated in tumor angiogenesis. The upregulation of SFRP2 in the tumor vasculature suggests a link between SFRP2 and angiogenesis. Courtwright et al. first described the pro-angiogenic effects of SFRP2 (99). *In vitro* experiments showed that SFRP2 promotes the survival and migration of endothelial cells. At the genetic level, SFRP2 treatment of endothelial cells upregulates pro-angiogenic genes such as VEGF-C (100). Another study suggested that recombinant SFRP2 treatment enhances angiogenesis in melanoma tumors, an effect that can be reversed by adding anti-SFRP2 antibodies (101). These data provide further evidence that the Wnt pathway plays a critical role in the pro-angiogenic effects of SFRP2. Peterson et al. further investigated this concept and found that the FzD5 receptor is crucial in the SFRP2-mediated Wnt signaling pathway (102). In endothelial cells lacking this receptor, intracellular calcium release is reduced, nuclear NFATc3 does not accumulate, and angiogenesis is impaired. In summary, the pro-angiogenic effect of SFRP2 largely depends on the non-canonical Wnt signaling pathway, potentially through direct binding to the FzD5 receptor on tumor endothelial cells. In this study, we identified high expression of SFRP2 in mural cells (especially pericytes) in AEGJ liver metastasis, suggesting that SFRP2 may influence the tumor microenvironment and liver metastasis by regulating pericyte function. The specific mechanism may involve high expression of SFRP2 in pericytes, promoting tumor angiogenesis and endothelial cell survival through the Wnt/Ca2+ signaling pathway, which contributes to AEGJ liver metastasis. However, the exact mechanism remains to be further investigated.

This study identifies tumor microenvironmental and molecular targets associated with liver metastasis in AEGJ, offering potential as novel therapeutic options. Translating these findings into effective therapeutic strategies requires further exploration of potential clinical applications and associated challenges. First, considering the roles of JUND, SFRP2, and other molecules in AEGJ hematogenous metastasis, developing targeted therapies against these molecules could offer new treatment options. For instance, designing specific inhibitors or gene regulation strategies to suppress SFRP2 expression in pericytes could mitigate its tumor-promoting effects within the microenvironment, thereby reducing metastatic potential. If further studies confirm stable SFRP2 expression across various patient subgroups, this molecular target could aid in patient stratification and individualized treatment planning, enhancing therapeutic precision. However, clinical translation of these targets presents several challenges. First, clinical validation of biomarkers is essential (103, 104).

Current findings of elevated SFRP2 expression need validation in larger patient cohorts to confirm its generalizability and specificity across populations. Furthermore, whether other molecules and pathways identified through single-cell sequencing possess therapeutic potential necessitates further *in vitro* and *in vivo* functional studies. Research involving specific cells may face technical challenges. For example, isolating tumor-associated pericytes presents significant technical challenges. Currently, most studies use flow cytometry (84, 85, 105, 106) or magnetic bead sorting (107) to isolate pericytes from tumor tissue and vasculature, though these methods often encounter issues with cell purity and cross-contamination. Some researchers propose microsurgical excision of tumor vasculature, followed by Matrigel encapsulation and a two-week culture to facilitate the natural migration of pericytes from the vessels (108, 109). While this method yields high-purity cells with minimal contamination, it is complex and time-consuming, suggesting areas for improvement. Without access to the relevant pericytes, molecular mechanism studies cannot progress, limiting conclusions to expression-level validation. Finally, the complexity of the AEGJ tumor microenvironment presents new challenges for clinical translation. Research indicates that diverse cell types and interactions within the microenvironment are critical to tumor progression and metastasis, and that the microenvironment consists of a complex, three-dimensional structure with multiple mechanisms (16, 51, 110–112). Therefore, future therapeutic strategies may require multi-target interventions, particularly combining targeted therapies with traditional treatments. Investigating these factors will be crucial for achieving comprehensive therapeutic outcomes.

Limitations of this study: We collected liver metastasis samples from AEGJ patients for joint analysis. However, due to limited experience in specimen collection, the liver metastasis samples were found to be necrotic tissue during preliminary analysis. As a result, they could not be included in the final single-cell transcriptome analysis. Consequently, our study could not analyze the metastatic process or the intrinsic molecular connections between the primary and liver metastasis sites in AEGJ. Furthermore, we only collected samples from two AEGJ liver metastasis patients who underwent surgery for single-cell sequencing analysis. The small sample size resulted in a low number of cells in several cell subpopulations. Our findings must be validated in a larger patient cohort.

## 5 Conclusion

In summary, our study revealed high expression of the SFRP2 gene in pericyte cells during the liver metastasis of adenocarcinoma of the esophagogastric junction (AEGJ) using 10X single-cell sequencing. Additionally, we identified a specific fibroblast subpopulation in AEGJ liver metastasis. These findings not only deepen our understanding of the mechanisms underlying AEGJ liver metastasis but also offer new insights and targets for future therapeutic strategies. Further research will aim to elucidate the specific functions and regulatory mechanisms of SFRP2 in pericytes, thus providing a theoretical foundation for precision therapy in AEGJ patients.

## Data Availability

The datasets presented in this study can be found in online repositories. The names of the repository/repositories and accession number(s) can be found below: https://ngdc.cncb.ac.cn/gsa-human/browse/HRA008269, HRA008269.

## Supplementary material

Supplementary Figure 1 A more detailed schematic diagram of single-cell clustering analysis. **(A)** UMAP plot showing the clustering of 22 cell subsets in AEGJ liver metastasis (EGJ-liver), AEGJ (EGJ), gastric body cancer (Body), gastric antrum (Antrum) and normal tissues (NC). **(B)** Proportions of 22 cell subsets in each group. **(C)** Dot plot showing representative top 3 marker genes in 22 cell subsets. **(D)** UMAP plot color-coded for expression of marker genes.

Supplementary Figure 2 Supplementary explanation of four significantly expression genes in fibroblast. **(A-D)** UMAP plot for the expression of IL24, JUND, S100A8 and S100A9 in different groups. **(E, F)** S100A8 and S100A9 genes expression in Stomach adenocarcinoma (STAD) in GEO database (GSE54129). **(G, H)** S100A8 and S100A9 expression and distribution in STAD-GSE1672972.

Supplementary Figure 3 Other 10 different expression genes and their combinational analysis. The results for these genes present the following questions: some genes show inconsistent expression between our results and TCGA data, and some genes have higher expression in liver metastasis but are associated with better survival in KM analysis, which is clearly contradictory.
