# Supplementary figures and images for: Integrated single-cell RNA sequencing reveals the tumor heterogeneity and microenvironment landscape during liver metastasis in adenocarcinoma of esophagogastric junction
Source: Front Immunol. 2025 Jan 9;15:1484234. doi: 10.3389/fimmu.2024.1484234 (PMC11754270; doi:10.3389/fimmu.2024.1484234)

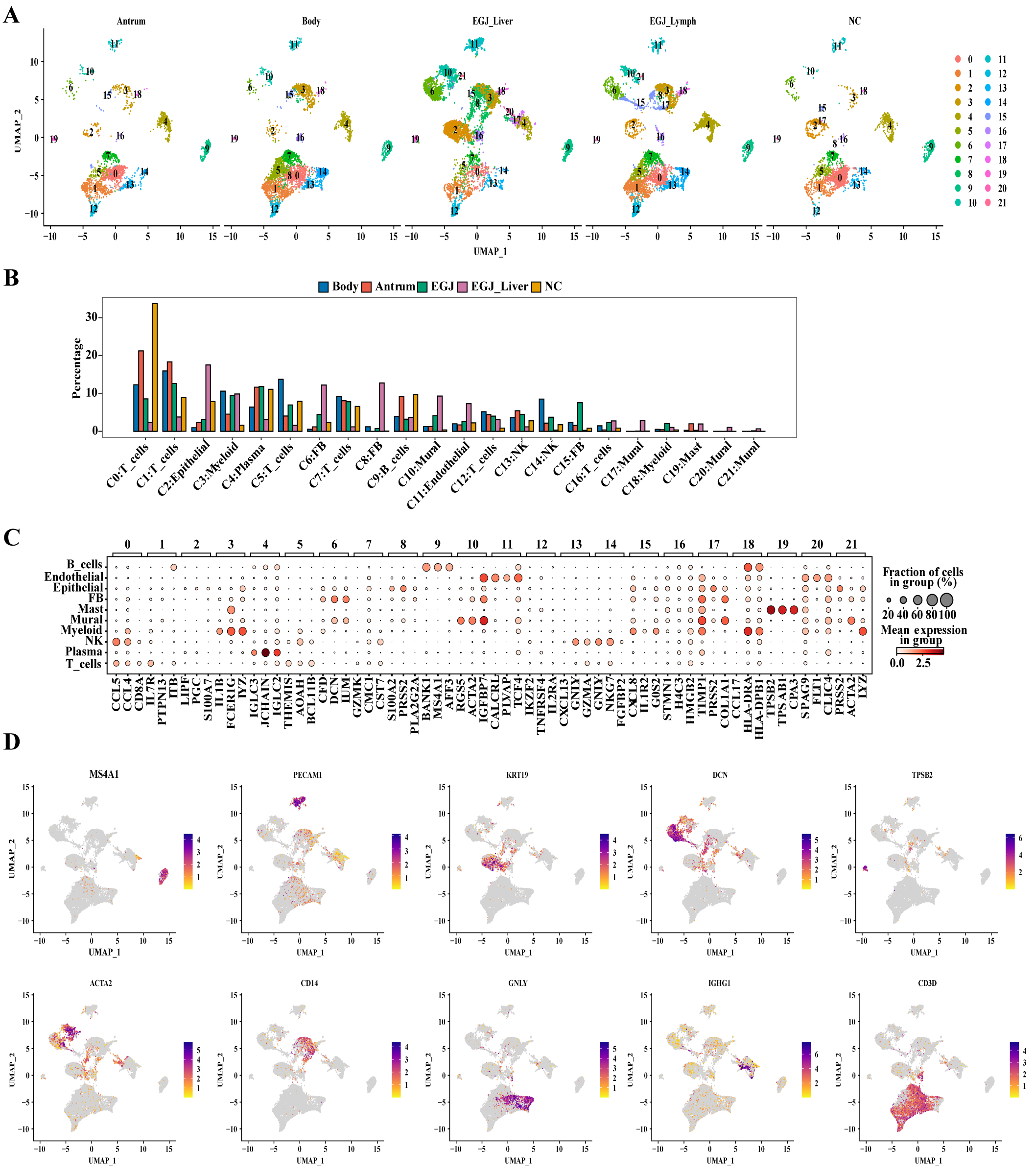

Supplement: Supplementary Figure 1 — A more detailed schematic diagram of single-cell clustering analysis. (A) UMAP plot showing the clustering of 22 cell subsets in AEGJ liver metastasis (EGJ-liver), AEGJ (EGJ), gastric body cancer (Body), gastric antrum (Antrum) and normal tissues (NC). (B) Proportions of 22 cell subsets in each group. (C) Dot plot showing representative top 3 marker genes in 22 cell subsets. (D) UMAP plot color-coded for expression of marker genes. [file Image1.tif]

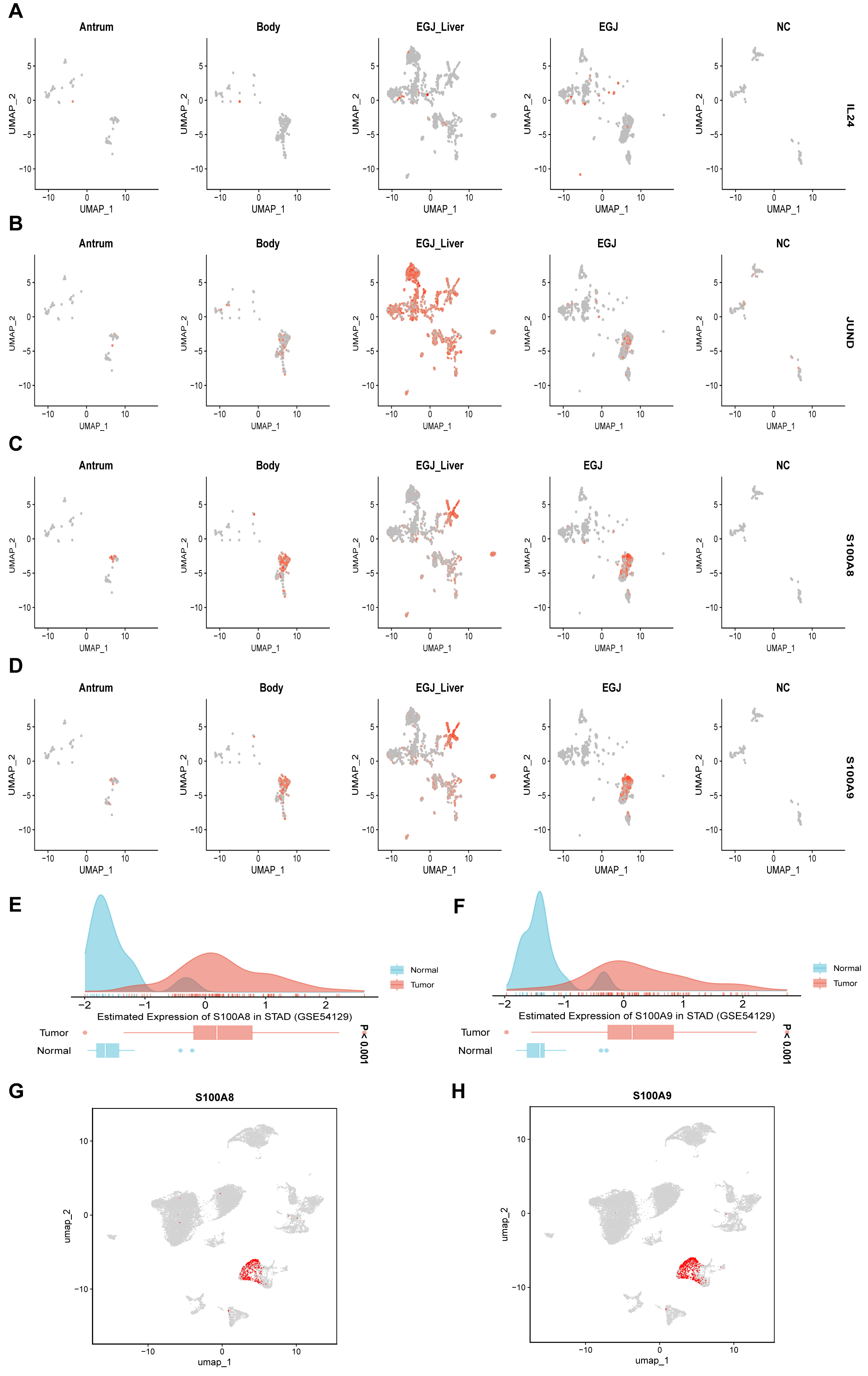

Supplement: Supplementary Figure 2 — Supplementary explanation of four significantly expression genes in fibroblast. (A-D) UMAP plot for the expression of IL24, JUND, S100A8 and S100A9 in different groups. (E, F) S100A8 and S100A9 genes expression in Stomach adenocarcinoma (STAD) in GEO database (GSE54129). (G, H) S100A8 and S100A9 expression and distribution in STAD-GSE1672972. [file Image2.tif]

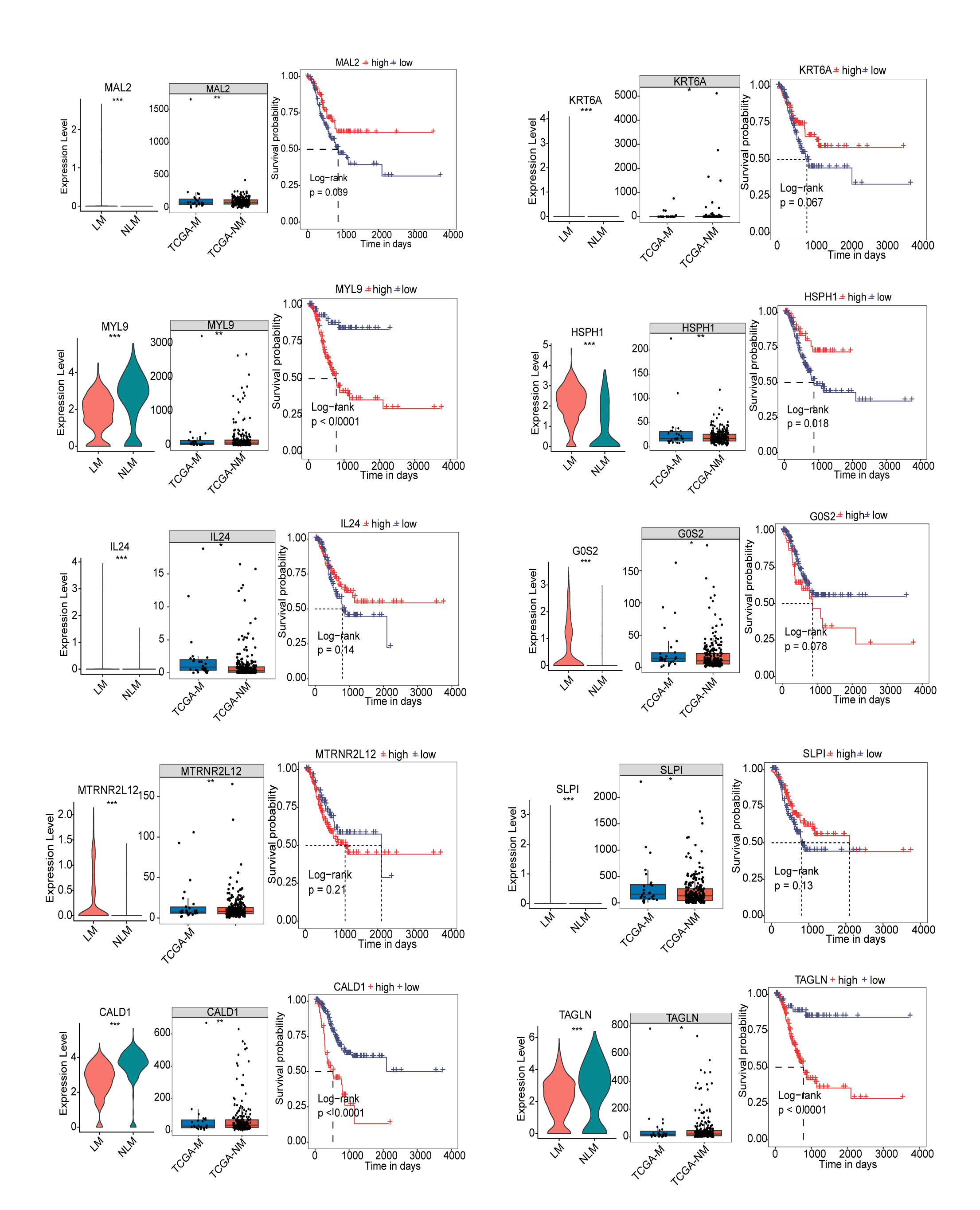

Supplement: Supplementary Figure 3 — Other 10 different expression genes and their combinational analysis. The results for these genes present the following questions: some genes show inconsistent expression between our results and TCGA data, and some genes have higher expression in liver metastasis but are associated with better survival in KM analysis, which is clearly contradictory. [file Image3.tif]
